# Biological Evaluation of Molecular Spherical Nucleic Acids: Targeting Tumors via a Hybridization-Based Folate Decoration

**DOI:** 10.1021/acsomega.4c10047

**Published:** 2025-02-04

**Authors:** Tatsiana Auchynnikava, Antti Äärelä, Olli Moisio, Heidi Liljenbäck, Putri Andriana, Imran Iqbal, Toni Laine, Senthil Palani, Jyrki Lehtimäki, Johan Rajander, Harri Salo, Anu J. Airaksinen, Pasi Virta, Anne Roivainen

**Affiliations:** † Turku PET Centre, University of Turku and Turku University Hospital, Turku FI-20520, Finland; ‡ Department of Chemistry, University of Turku, Turku FI-20500, Finland; § Research and Development, Orion Pharma, Turku FI-20380, Finland; ∥ Turku Center for Disease Modeling, University of Turku, Turku FI-20520, Finland; ⊥ Turku PET Centre, Accelerator Laboratory, Åbo Akademi University, Turku FI-20520, Finland; # InFLAMES Research Flagship, University of Turku, Turku FI-20520, Finland

## Abstract

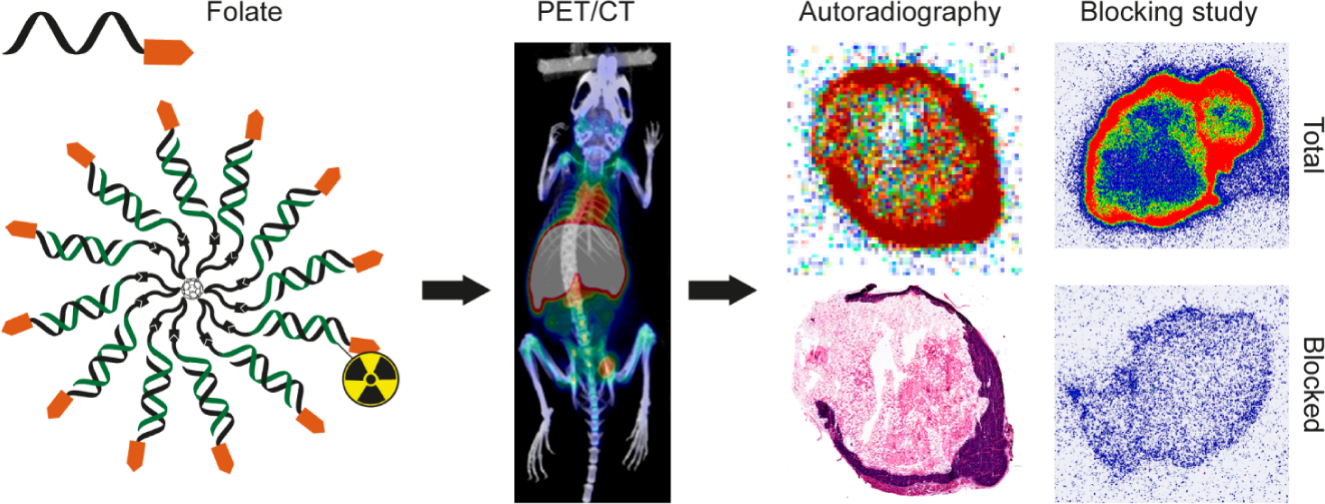

Folate receptors (FRs), membrane-bound proteins that bind specifically to folate with high affinity, are overexpressed by various cancer types and are therefore used as targets for delivery of therapeutic agents. Molecular spherical nucleic acids (MSNAs) are dendritic formulations of oligonucleotides (ONs) that may have advantages over linear parent ONs with respect to delivery properties. Here, we assembled folate-decorated MSNAs, site-specifically radiolabeled them, and then biologically evaluated their effects in mice bearing HCC1954 breast cancer xenograft tumors. The biodistribution of intravenously administered ^18^F-radiolabeled MSNAs was monitored using positron emission tomography/computed tomography imaging. The results revealed higher accumulation of folate-decorated MSNAs in FR-expressing organs such as the liver, kidney, and spleen, as well as a higher tumor-to-muscle ratio than that observed for MSNAs without the folate decoration. However, the observed increase was statistically significant only for MSNA structures with a PO backbone. The observed selective uptake of folate-decorated MSNAs highlights their potential as targeted delivery vehicles for therapeutic and diagnostic agents in FR-overexpressing cancers.

## 1 Introduction

Spherical nucleic acids (SNAs) are oligonucleotide (ON) nanoparticles comprising a core material (e.g., gold, silica, liposomes, or proteins) surrounded by a radially arranged layer of ONs.^1−4^ SNAs possess characteristics that can be harnessed for delivery of therapeutic ONs; such characteristics overcome some major hurdles associated with ONs.^5−7^ SNAs are taken up effectively by various cell types via class A scavenger receptor-mediated endocytosis, a process influenced by the density and chemical composition of the ONs.^8−10^ SNAs are also resistant to degradation by nucleases^11,12^ and can evade renal clearance, although this depends on their size and ability to form a protein corona.^13,14^ Cell- or tissue-specific ligands can be hybridized to the outer surface of SNAs, a process that may mask unfavorable biodistribution properties associated with the loaded ONs.^15,16^ In parallel with polydisperse SNAs, atomically uniform molecular spherical nucleic acids (MSNAs),^2,17^ other dendritic ONs,^18^ and their conjugates with cell-specific ligands^15,19^ have received increasing interest as potential delivery vehicles.

Folate, also known as vitamin B9, is an essential nutrient involved in cell metabolism and in DNA synthesis and repair.^20,21^ Folate receptors (FRs) are membrane-bound proteins that bind specifically to folate with high affinity.^22^ Many cancer cells, including ovarian, breast, lung, and colorectal cancer cells, exhibit increased surface expression of FRs, making them ideal targets for folate-based drug delivery systems.^23−27^

Previous reports show that FR-targeted delivery can be used to deliver ONs and other nanostructures to malignant tissues, and that they have several advantages over nontargeted delivery methods.^28−34^ The high specificity of FR targeting reduces exposure of healthy tissues to potent drugs, resulting in reduced systemic toxicity and improved patient tolerability.^35^ FR-targeted nanoparticles evade clearance by blood monocytes or macrophages within the reticuloendothelial system,^36^ leading to a prolonged circulation time and accumulation at the target site. Additionally, FR-targeted delivery vehicles can access the central nervous system through FR-mediated transcytosis.^37^

The increasing number of beneficial therapeutic delivery characteristics of MSNAs suggests that coating with folate may increase the precision with which therapeutic ONs are delivered to cancerous tissues. To evaluate these characteristics *in vivo*, MSNAs can be radiolabeled prior to analysis by preclinical positron emission tomography (PET). Recently, click chemistry, particularly the inverse electron demand Diels–Alder (IEDDA) reaction between radiolabeled tetrazine (Tz) and *trans-*cyclooctene (TCO)-functionalized targeting nanoparticles, has gained much attention due to its fast reaction rates, versatility, and mild reaction conditions.^38−43^ Our previous studies show that the IEDDA reaction between [^18^F]FDG-Tz and TCO-functionalized MSNA is highly efficient, generates product at a high yield, and is reproducible, without requiring harsh conditions.^44^ In addition, it brings the potential for therapeutic and theranostic approaches. The combination of imaging and therapy provides a theranostic approach that allows real-time monitoring of treatment efficacy and the ability to personalize interventions based on tumor response.

**Scheme 1:**
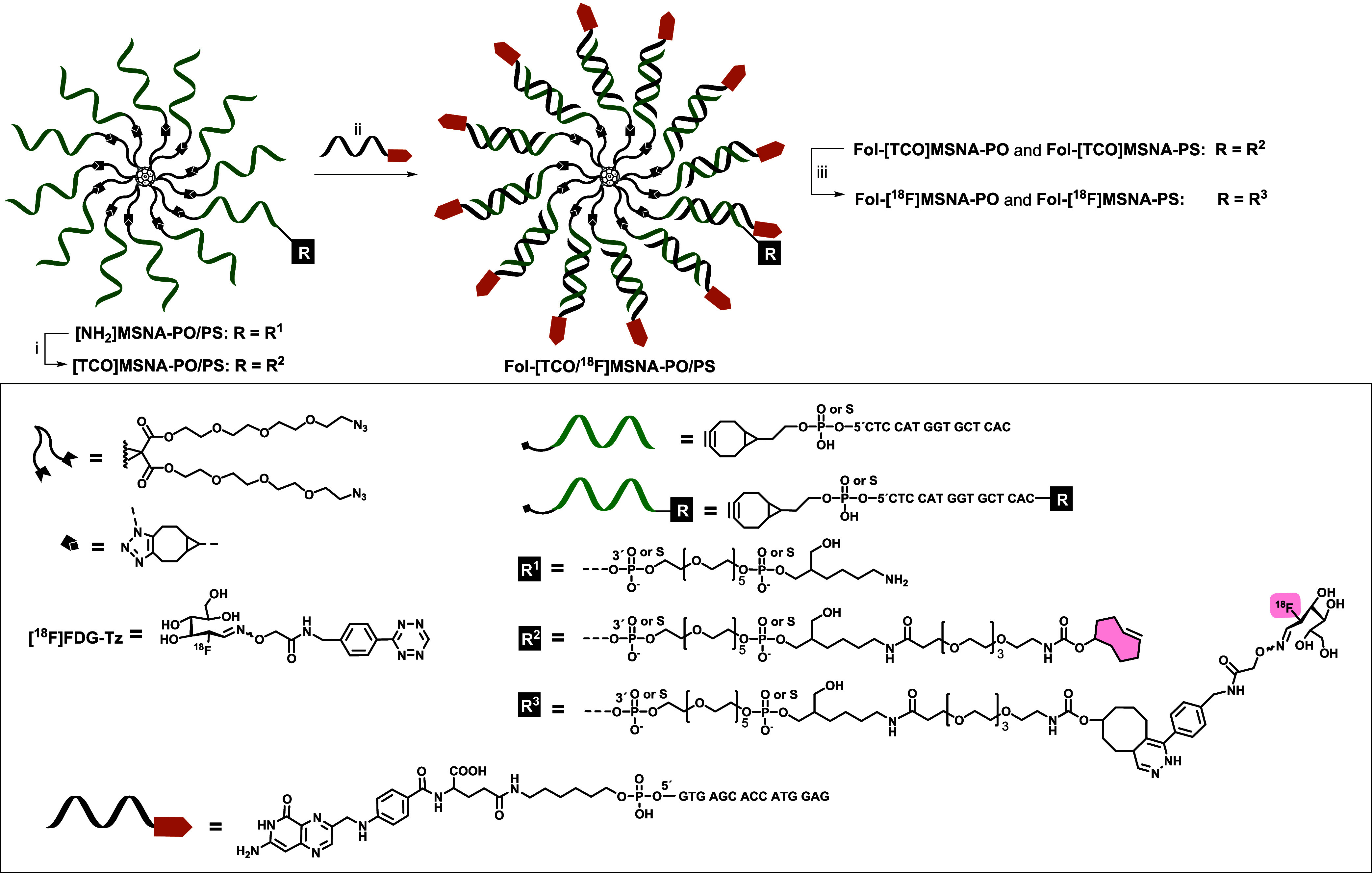
Synthesis of Radiolabeled MSNAs The reaction conditions were as follows: (i) **TCO-PEG4-NHS** in 0.1 M sodium borate (pH 8.5), 4 h at room temperature (rt); (ii) folate-decorated **Fol-ON** (12 eq.) in PBS (pH 7.4), 1 h at rt; (iii) **[**^**18**^**F]FDG-Tz** in PBS (pH 7.4), 5 min at rt.

Herein, [60]fullerene-based MSNAs comprising an antisense ON sequence with either a native phosphodiester (MSNA-PO)^44^ or a phosphorothioate (MSNA-PS)^44^ backbone were generated against human epidermal growth factor receptor 2 (HER2) mRNA and then coated with folate. PS backbone modification was chosen due to previously reported increased *in vivo* stability.^45,46^ Hybridization-mediated constructs harboring folate-conjugated ONs complementary to the anti-HER2 sequence of MSNAs were then added. These folate-decorated MSNAs (**Fol-[TCO]MSNA-PO** and **Fol-[TCO]MSNA-PS**) were monolabeled specifically with fluorine-18 (Scheme 1) and then administered intravenously (i.v.) into HCC1954 tumor-bearing mice via the tail vein. Finally, the mice were imaged by using PET/computed tomography (CT). We also showed the presence of FRs in HCC1954 breast cancer xenograft tumor cells. This study aims to explore whether folate decoration enhances the uptake and improves the targeting capabilities of MSNAs by using the high affinity of the folate receptors for folate. By directing MSNAs toward FR-expressing tumors, we anticipate improving targeting specificity and higher accumulation in the tumor compared with previously reported structures, thus demonstrating the potential of folate-decorated MSNAs as an advanced platform for cancer imaging and therapy.

## 2 Materials and Methods

### 2.1 Synthesis of Folate-Modified Fol-ON

Folate *N*-hydroxysuccinimidyl ester (1.5 μmol in 5 μL of dimethyl sulfoxide, DMSO) was added to a buffered mixture of 5′-amino modified phosphodiester oligonucleotide bearing the HER2-sense sequence (50 nmol in 100 μL of 0.1 M sodium borate, pH 8.5). The reaction mixture was shaken gently for 4 h at room temperature (rt) and then subjected to reversed-phase high-performance liquid chromatography (RP-HPLC). An analytical RP column (Thermo Scientific Hypersil ODS C18, 250 × 4.6 mm, 5 μm), a linear gradient from 5% to 45% acetonitrile in 50 mmol L^–1^ triethylammonium acetate over 25 min, a flow rate of 1.0 mL min^–1^, and detection at 260 nm were used for purification. The product fractions were collected and lyophilized to dryness. The authenticity of the product was verified by electrospray ionization time-of-flight mass spectrometry (MS ESI-TOF) (Figure S1). The yield (25%) of the isolated product (i.e., **Fol-ON)** was determined by the UV absorbance at 260 nm.

### 2.2 Synthesis of Hybridization-Based Folate Conjugates Fol-[TCO]MSNA-PO and Fol-[TCO]MSNA-PS

TCO-functionalized PO and PS MSNAs were prepared according to a previously published protocol.^44^ Briefly, the azide-modified C_60_-core (0.8 μmol in 900 μL of DMSO) was treated with a 5′-bicyclo[6.1.0]non-4-yne (BCN)- and 3′-amino-modified ONs (0.2 μmol in 100 μL of H_2_O) to yield monofunctionalized C_60_-ON conjugates, which were then isolated by RP-HPLC and exposed to a slight excess of 5′-BCN-modified ONs (1.2 eq./azide group) in an aqueous solution containing 1.5 M NaCl. This process yielded 12-arm MSNAs with an amino modification, specifically **[NH**_**2**_**]MSNA-PO** and [**NH**_**2**_**]MSNA-PS**; these were then TCO-functionalized through selective amide coupling using **TCO-PEG4-NHS** (50 equiv) in 0.1 M sodium borate, pH 8.5. After 4 h reaction at rt, phosphate-buffered saline (PBS) was added and the excess **TCO-PEG4-NHS** was removed by centrifugal filtration for 9 min at 14,000× *g* (Amicon Ultra, 30-kDa molecular weight cutoff; Merck, Darmstadt, Germany). PBS addition and centrifugation were repeated five times to isolate the desired [TCO]-MSNAs in 95% yield. To add the folate decoration, **[TCO]MSNA-PO** or **[TCO]MSNA-PO** (8 nmol in 30 μL of phosphate-buffered saline, PBS) was treated with complementary **Fol-ONs** (96 nmol in 100 μL of PBS) for 1 h at rt. The authenticity and homogeneity of **Fol-[TCO]MSNA-PO** and **Fol-[TCO]MSNA-PS** were verified using a size-exclusion chromatography apparatus equipped with a multiple angle light scattering detector (SEC-MALS) (Figures S2 and S3) and by polyacrylamide gel electrophoresis (PAGE) (Figure S4). PAGE, SEC-MALS, and enzymatic stability of the MSNAs were performed, and detailed experimental procedures are described in the Supporting Information.

### 2.3 Radiosynthesis of [^18^F]Fol-MSNAs

**[**^**18**^**F]FDG-Tz** was synthesized as described earlier.^47^ Briefly, a commercially available tetra-*O*-acetyl mannose triflate (Sigma-Aldrich, St. Louis, MO, USA) was used as the starting material to generate 2-[^18^F]fluoro-2-deoxy-*D*-glucose ([^18^F]FDG). After purification by semipreparative HPLC to remove excess precursor, [^18^F]FDG was conjugated to *N*-(4-(1,2,4,5-tetrazin-3-yl)benzyl)-2-(aminooxy)acetamide via oxime formation. After semipreparative HPLC purification, [^18^F]FDG-Tz (49.6 ± 3.8 μL in PBS [pH 7.4], *n* = 3; 1.0 nmol, 119.8 MBq, *n* = 2) was mixed with **Fol-[TCO]MSNA-PO** or **Fol-[TCO]MSNA-PS** (9.3 ± 0.9 nmol in 37–97 μL; *n* = 3). The IEDDA click reaction between radiolabeled Tz and TCO-functionalized MSNA was conducted according to a previously published method.^44^ Briefly, the reaction was maintained for 5 min at rt, and the reaction products were purified by ultrafiltration (Amicon Ultra filter devices; 0.5 mL, 30 kDa cutoff; Merck, Darmstadt, Germany). For this, the reaction mixture was placed into the filter device, which was then filled with RNase-free PBS until the volume reached 200 μL. The filter was centrifuged three times at 14,100*g* for 5 min at rt. The final formulation in RNase-free PBS was subjected to radio-SEC (Waters Protein-Pak; 0.1 M monopotassium phosphate, pH 7.0, 1 mL/min) and radio-TLC (thin-layer chromatography).

### 2.4 HER2 and FR-α Levels in HCC1954 Breast Cancer Cells

To quantify folate receptor-alpha (FR-α) and HER2 levels, human HCC1954 ductal breast carcinoma cells (American Type Culture Collection, Manassas, VA, USA) were cultured in RPMI-1640 medium (Gibco/Thermo Fisher Scientific, Waltham, MA, USA) supplemented with 10% fetal bovine serum (Biowest, Nuaillé, France) and 2 mmol of l-glutamine (GlutaMax 100x, Gibco/Thermo Fisher Scientific, Waltham, MA, USA). Cells were then harvested and incubated with phycoerythrin (PE)-conjugated antihuman FR-α antibody (mouse IgG2a, catalog number 908303, BioLegend, San Diego, CA, USA), Alexa-488-conjugated antihuman HER2 antibody (human IgG1-Alexa-488, catalog number 570451, BD Biosciences, Franklin Lakes, NJ, USA) or isotype control antibodies (mouse IgG2a-PE, catalog number 400212, BioLegend, and mouse IgG1-Alexa-488, catalog number 570390, BD Biosciences, Franklin Lakes, NJ, USA). After washing, cells were fixed with paraformaldehyde and flow cytometry was performed with BD-Fortessa device (BD Biosciences, Franklin Lakes, NJ, USA) and analyzed with Flowing Software (Cell Imaging and Cytometry Core, Turku Bioscience, Turku, Finland) (Figure S6).

### 2.5 Animal Experiments

All animal experiments were performed in compliance with the European Union Directive 2010/EU/63 on the protection of animals used for scientific purposes and were approved by the National Project Authorization Board in Finland (license number: ESAVI/21485/2020). Human HCC1954 ductal breast carcinoma cells (American Type Culture Collection, Manassas, VA, USA) and female Rj:Athymic-*FOXn1nu/nu* mice (Janvier Laboratories, Le Genest-Saint-Isle, France) were prepared as described previously.^44^ Animals (aged 6–8 weeks) were inoculated subcutaneously into the upper back region with 5 million cells suspended in 50% nonsupplemented RPMI-1640 (Gibco product, Waltham, MA, USA) and 50% Matrigel (Corning, Corning, NY, USA). Over a span of 3–6 weeks, tumor growth was observed visually, and mouse body weight was measured on a weekly basis. The animals were kept in individually ventilated cages under standardized specific pathogen-free conditions at the Central Animal Laboratory, University of Turku under a 12 h light/dark cycle. All mice had access to standard soy-free food and tap water *ad libitum*.

### 2.6 Biological Evaluation and Image Analysis

Tumor-bearing mice were injected i.v. with **[**^**18**^**F]MSNA-PO, [**^**18**^**F]MSNA-PS, [**^**18**^**F]Fol-MSNA-PO,** or **[**^**18**^**F]Fol-MSNA-PS** (6.4 ± 1.6 MBq in 35–110 μL, *n* = 23) at the start of a 60 min dynamic PET study conducted using an Inveon Multimodality PET/CT (Siemens Medical Solutions, Knoxville, TN, USA). The obtained data were reconstructed using an ordered subsets expectation maximization three-dimensional algorithm divided into 6 × 10, 4 × 60, and 11 × 300 s time frames. Carimas software (version 2.10, Turku PET Center, Turku, Finland) was used for image analysis. CT scans were utilized as an anatomical reference and fused with PET images to guide the definition of regions of interest (ROIs) in the tumor, muscle (skeletal), blood pool (heart left ventricle cavity), kidneys, liver, and urinary bladder (urine content). Tumor ROIs were drawn over viable tumor tissue, excluding the necrotic core. The process was described in detail previously.^47^ PET data are represented as the standardized uptake value (SUV) as a function of time postinjection. Inveon Research Workplace 4.1 software was used to capture representative PET/CT images.

Following the PET/CT scan, animals were euthanized, and organs of interest were collected for weighing and measuring in a γ-counter (Triathler, Hidex, Turku, Finland). The results are expressed as the injected radioactivity dose per gram of tissue (%ID/g). Tumors were cryosectioned (20 μm) for autoradiography and hematoxylin-eosin (H&E) staining. For autoradiography, cryosections were opposed to phosphor imaging plates (BAS-TR2025, Fuji Photo Film Co. Ltd., Tokyo, Japan) overnight, which were then scanned with a BAS-5000 device (Fuji Photo Film Co. Ltd., Tokyo, Japan), and analyzed with an AIDA Image Analyzer v.4.19 (Raytest Isotopenmessgeräte, Straubenhardt, Germany). Tumor sections were stained with H&E according to standard procedure after being fixed with 10% formalin (phosphate buffered, including formaldehyde 4%). Stained sections were scanned with a digital slide scanner (Pannoramic P1000; 3DHistec Ltd., Budapest, Hungary).

### 2.7 *In Vitro* Blocking Study

The specificity of folate-decorated MSNA for FRs in tumors was investigated as previously described.^48^ Briefly, 20 μm cryosections of tumor (three sections from three different tumors) were defrosted gently, first at 4 °C and then at rt. After preincubation in PBS for 20 min at rt, sets of slides were incubated for 30 min at rt with **[**^**18**^**F]FOL**, a tracer that binds to FRs.^49^ Another set of slides was preincubated for 10 min with a molar excess of **Fol-[TCO]MSNA-PO** (1 μM) as a blocking agent prior to the application of **[**^**18**^**F]FOL**. The slides were then rinsed with cold PBS and dipped into cold water. After drying, the slides were exposed to phosphor imaging plates (BAS-TR2025, Fuji Photo Film Co. Ltd., Tokyo, Japan) for approximately 4 h (at least 2 physical half-lives of ^18^F) and scanned with a Fujifilm BAS-5000 scanner (Fujifilm, Tokyo, Japan). The results were analyzed with Carimas software and expressed as the amount of photostimulated luminescence per square millimeter (PSL/mm^2^).

### 2.8 Statistical Analysis

GraphPad Prism (version 9.1.1) was used for all statistical analyses and the graphical presentation of data. The outcomes of the biological evaluation are expressed as the mean ± standard deviation (s.d.). Statistical significance was determined through application of multiple unpaired *t*-tests, and *p < 0.05, **p < 0.01, ***p < 0.001, ****p < 0.0001 were considered significant.

## 3 Results

### 3.1 Synthesis and Characterization of TCO-Modified Folate-Decorated MSNAs

**Figure 1:**
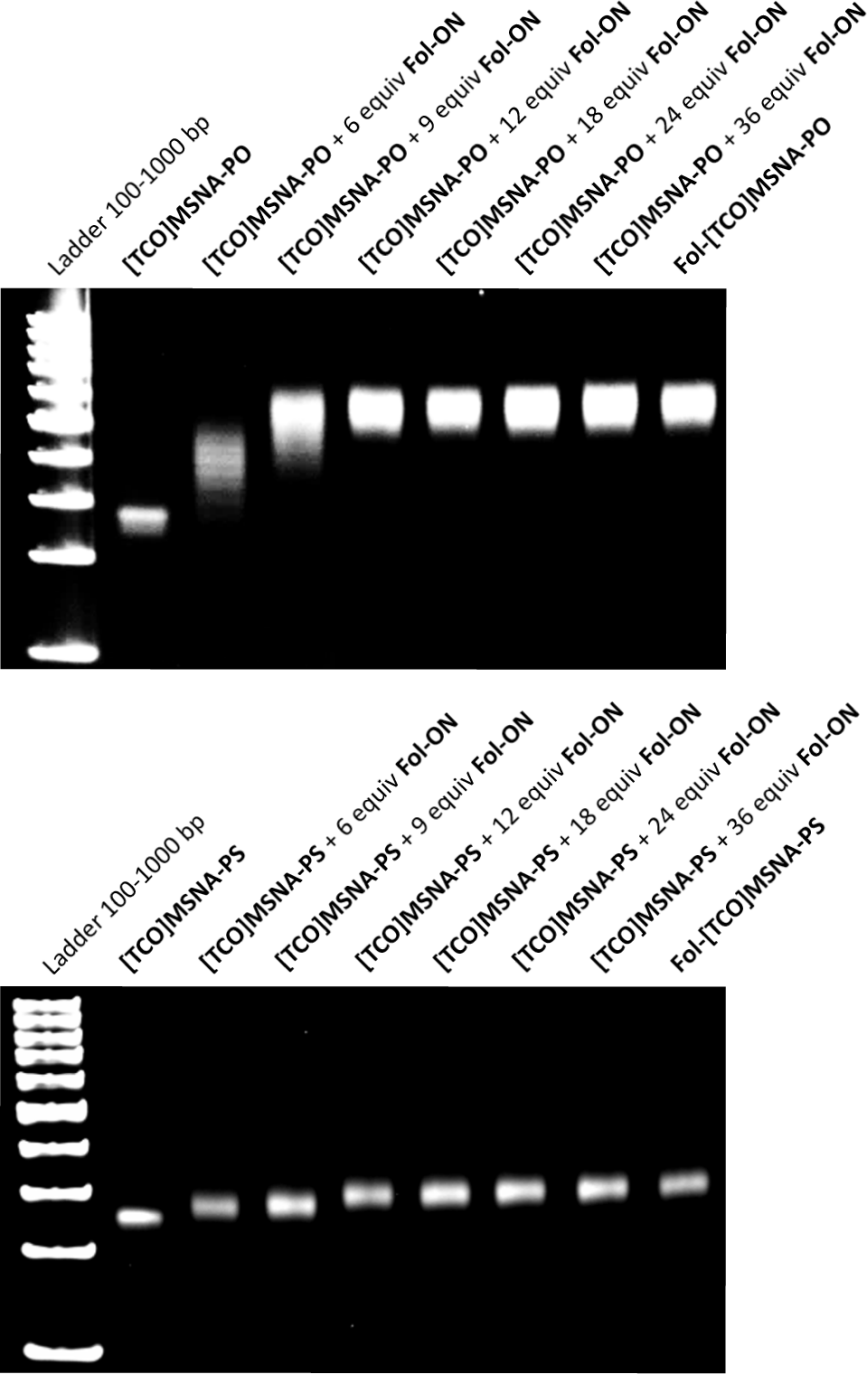
PAGE analysis of folate-decorated MSNAs after hybridization.

The TCO-modified MSNAs used for hybridization-based folate decoration were prepared as described previously.^44^**[NH**_**2**_**]MSNA-PO** and **[NH**_**2**_**]MSNA-PS** were isolated with a yield of 30–45%. These [NH_2_]MSNAs were then TCO-functionalized with TCO-PEG_4_-NHS, and **[TCO]MSNA-PO** and **[TCO]MSNA-PS** were isolated by centrifugal filtration (95% recovery). Next, **[TCO]MSNA-PO** and **[TCO]MSNA-PS** were mixed with 6–36 equiv of **Fol-ON** in PBS (pH 7.4), slower-migrating broad bands were observed on the gel, indicating the formation of hybridization-mediated MSNAs **Fol-[TCO]MSNA-PO** and **Fol-[TCO]MSNA-PS** (Figure 1). For both **[TCO]MSNA-PO** and **[TCO]MSNA-PS**, increasing the amount of **Fol-ON** beyond 12 equiv did not affect gel migration, indicating that complete hybridization is achieved already with 12 equiv. Interestingly, the hybridization conjugates of **[TCO]MSNA-PS** migrated markedly further than those of **[TCO]MSNA-PO**. To prepare folate-decorated MSNAs for the radiolabeling and biodistribution experiments, **[TCO]MSNA-PO** and **[TCO]MSNA-PS** were treated with 12 equiv of **Fol-ON** in PBS at r.t. for 1 h. SEC-MALS was used to evaluate the homogeneity and molecular weights of the MSNAs (Figures S2 and S3). The major peaks in the chromatograms represented the MSNAs. In addition, faster eluting fractions, representing noncovalent aggregates, were observed. This is a common occurrence with macromolecular analytes in SEC-MALS. The MALS-based detection of the major peaks revealed molecular masses: **Fol-[TCO]MSNA-PO** = 114.9 ± 0.1 kDa (Figure S2) and **Fol-[TCO]MSNA-PS** = 124.9 ± 0.1 kDa (Figure S3), which represented the MSNAs (calculated molecular masses: 124.1 and 126.9 kDa, respectively) with relatively large errors (ca. 9 and 2 kDa, respectively). However, the MALS-based analysis of molecular masses is not necessarily accurate with these size nucleic acid constructs (being also dependent on the metal ion content), and the observed values matched sufficiently well with the calculated ones.

### 3.2 Synthesis and Characterization of ^18^F-Labeled MSNAs

**[**^**18**^**F]FDG-Tz** used for the IEDDA reaction with TCO-functionalized MSNAs was synthesized in two steps. Initially, **[**^**18**^**F]FDG** was produced from commercially available mannose triflate, followed by an oxime formation step to generate **[**^**18**^**F]FDG-Tz**. Two rounds of semipreparative HPLC were conducted to remove excess mannose triflate and the Tz precursor.^47,50^

The IEDDA reaction between **[**^**18**^**F]FDG-Tz** and folate-decorated MSNAs was completed in just 5 min at r.t., resulting in high yields (82.6 ± 15.7%, *n* = 3) and excellent radiochemical purity (RCP > 99% by radio-SEC and radio-TLC, Figure S7). The molecular weights of radiolabeled **Fol-[TCO]MSNA-PO** and **Fol-[TCO]MSNA-PS** extracted from SEC-MALS were 113.8 ± 0.1 kDa (calculated 124.5 kDa) and 139.1 ± 2.3 kDa (calculated 127.3 kDa), respectively (Figures S8 and S9). PAGE analysis (Figure S4) revealed that the radiolabeled MSNAs yielded bands similar to those of their unlabeled precursors, which in conjunction with SEC-MALS analysis indicates that the selected radiolabeling protocol does not compromise the structural integrity of the double helical MSNAs.

### 3.3 Enzymatic Stability of the MSNAs

MSNAs (**[TCO]MSNA-PO/PS** and **Fol-[TCO]MSNA-PO/PS**) were incubated in a Tris buffer (pH 7.5) containing DNase I (1 U/nmol of effective oligonucleotide concentration) and the enzyme-catalyzed degradation was monitored by PAGE. As expected, [**TCO]MSNA-PS** (consisting of the phosphorothioate backbone) proved mainly intact in the given conditions, whereas [**TCO]MSNA-PO** and its hybridization complex **Fol-[TCO]MSNA-PO** (both consisting of the native phosphodiester backbone) underwent rapid and complete DNase I-catalyzed cleavage. On **Fol-[TCO]MSNA-PS**, in turn, degradation of the hybridized Fol-sens-HER2-strand resulted in virtually intact [**TCO]MSNA-PS**, as monitored by PAGE (Figure S5).

### 3.4 HER2 and FR-α Levels in HCC1954 Breast Cancer Cells

Flow cytometry analysis confirmed that HER2 and FR-α were predominantly expressed in HCC1954 cells (Figure S6). The expression levels of HER2 and FR-α were 98.0 ± 1.5% and 63.3 ± 8.1%, respectively.

### 3.5 Biological Evaluation

To evaluate the biodistribution of i.v.-injected ^18^F-labeled folate-decorated MSNAs, we performed dynamic PET/CT imaging and *ex vivo* studies in tumor-bearing mice and compared the results with those obtained for previously studied nonfolate radiolabeled MSNAs.^43^

**Figure 2:**
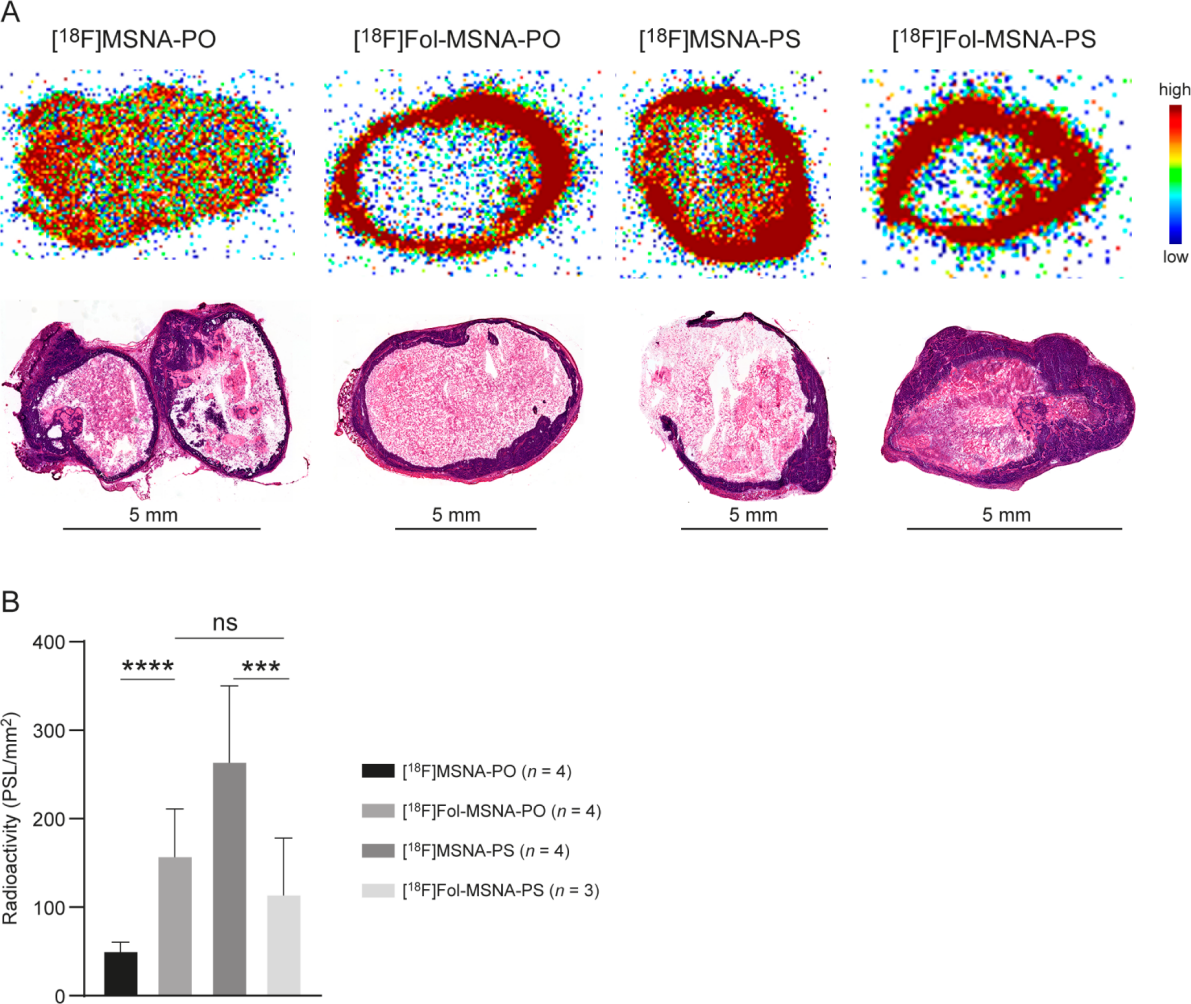
Autoradiography of HCC1954 tumor cryosections 60 min after intravenous injection of ^18^F-labeled MSNAs into female mice. (A) The amount of radioactivity (upper panels) in the viable tissue is clearly higher than that in the fluid-filled necrotic core, a finding confirmed by H&E staining (lower panels). Viable tumor tissue appears blue, while necrotic cells stain pink. (B) Quantification of the radioactive bands reveals differences between the ^18^F-labeled MSNAs. **[**^**18**^**F]MSNA-PO** and **[**^**18**^**F]MSNA-PS** data are reproduced from a previously published paper.^44^ PSL/mm^2^ = photostimulated luminescence per square millimeter. ***p < 0.001, ****p < 0.0001.

**Figure 3:**
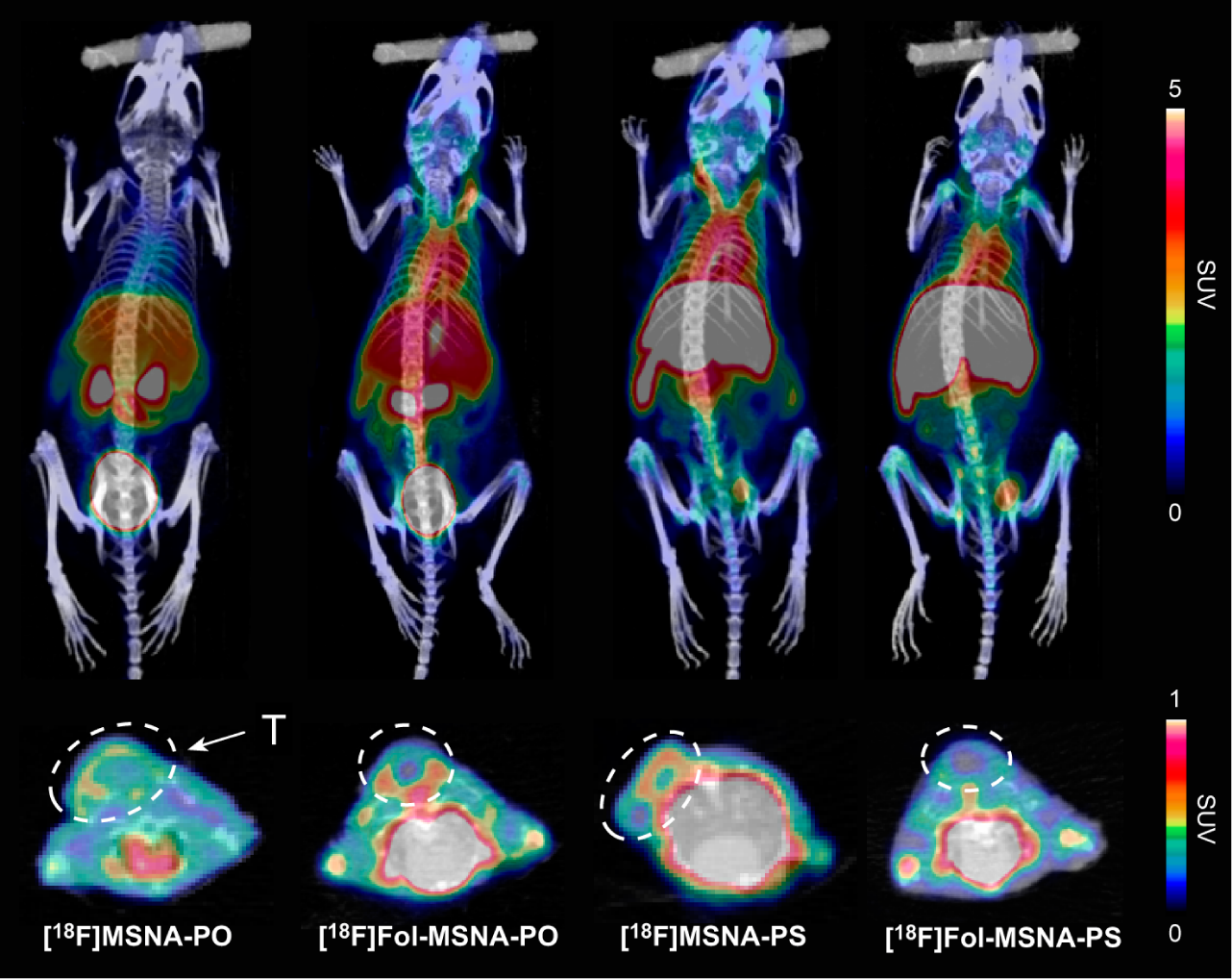
PET/CT images (maximum intensity projections in the coronal (upper panels) and transaxial plane (lower panels)) at 15–60 min postinjection of ^18^F-labeled MSNAs in HCC1954 tumor-bearing female mice. T denotes the tumor. **[**^**18**^**F]MSNA-PO** and **[**^**18**^**F]MSNA-PS** data are reproduced from a previously published paper.^44^ SUV = standardized uptake value.

Most of the tumors had a necrotic, fluid-filled core, lacking blood circulation, which was evident on both *ex vivo* autoradiography and H&E staining (Figures 2A and S10) and on *in vivo* PET/CT images (Figure 3). Otherwise, autoradiography indicated a homogeneous distribution of all tracers within the viable tumor tissue.

**Figure 4:**
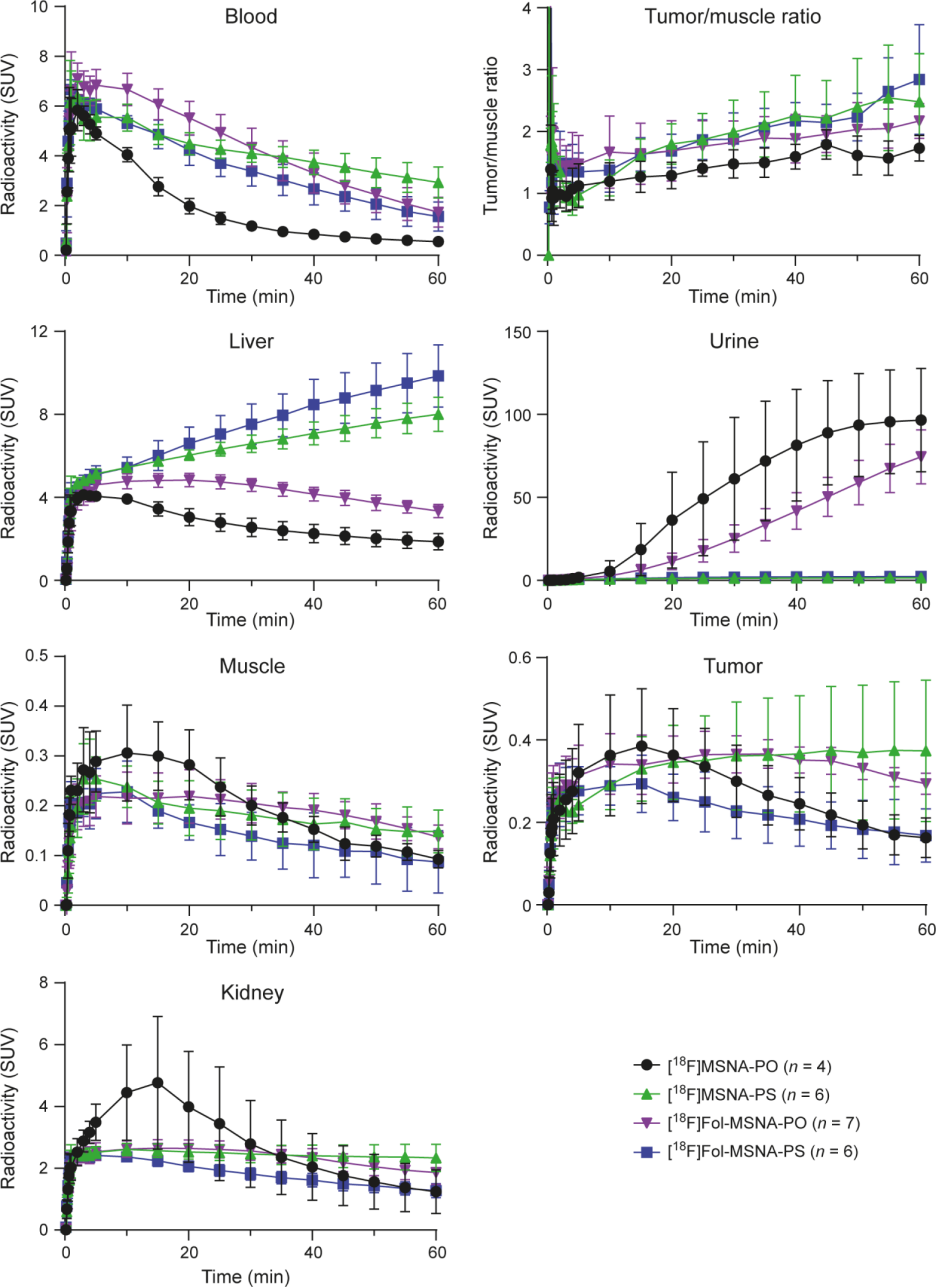
Time-activity curves of ^18^F-labeled MSNAs in HCC1954 tumor-bearing female mice (data were extracted from 60 min dynamic PET images). **[**^**18**^**F]MSNA-PO** and **[**^**18**^**F]MSNA-PS** data are reproduced from a previously published paper.^44^ SUV = standardized uptake value.

Folate decoration increased the tumor-to-muscle ratio of both **[**^**18**^**F]Fol-MSNA-PO** and **[**^**18**^**F]Fol-MSNA-PS** when compared with that of nonfolate structures, but the difference was only significant for **[**^**18**^**F]Fol-MSNA-PO** (Table S1). The tumor-to-muscle ratio was highest after **[**^**18**^**F]Fol-MSNA-PS** injection (2.84 ± 0.89), with an increasing trend toward the end of the 60 min scan (Figure 4).

Folate decoration increased the uptake of **[**^**18**^**F]Fol-MSNA-PO** significantly in several tissues when compared with the no-folate structure, with the largest differences observed in the spleen, bone marrow, ovaries, skin, adrenal glands, large intestine, kidneys, lungs, and liver (Table 1, Figure S11). *In vivo* PET/CT revealed that liver uptake of **[**^**18**^**F]Fol-MSNA-PS** was significantly higher than that of nonfolate MSNA (SUV 9.85 ± 1.50 vs 8.01 ± 0.83, *p* = 0.031, respectively), but no difference was observed upon *ex vivo* gamma counting. In addition, the kidney uptake of **[**^**18**^**F]Fol-MSNA-PS** tended to be higher (albeit not significantly). Interestingly, the amount of radioactivity in blood was significantly higher after folate decoration of MSNA-PO and significantly lower after folate decoration of MSNA-PS (Table 1). Such patterns were also observed after *in vivo* PET/CT and *ex vivo* autoradiography (Figures 2B and 4). The *ex vivo* %ID/g tumor results (Table 1) are likely not representative due to measurements being made in whole tumors, which include the necrotic, fluid-filled core.

**Table 1:** *Ex Vivo* Biodistribution (%ID/g) of ^18^F-Labeled MSNAs in Female HCC1954 Tumor-Bearing Mice at 60 Min Postintravenous Administration^a^^b^

Tissue	[^18^F]MSNA-PO	[^18^F]Fol-MSNA-PO	*p* value	[^18^F]MSNA-PS	[^18^F]Fol-MSNA-PS	*p* value	*p* value^b^
Adrenal gland	2.59 ± 0.15	4.05 ± 0.89	0.004	14.47 ± 3.30	19.48 ± 15.66	0.519	0.092
Blood	2.83 ± 0.40	7.21 ± 3.33	0.013	13.95 ± 4.01	4.42 ± 2.10	0.001	0.123
Bone (skull)	0.63 ± 0.14	0.95 ± 0.25	0.024	2.73 ± 1.13	1.74 ± 0.44	0.092	0.011
Bone + marrow (femur)	1.19 ± 0.05	2.55 ± 0.41	0.0001	5.69 ± 2.35	7.62 ± 1.02	0.113	0.0002
Brain	0.06 ± 0.01	0.10 ± 0.04	0.045	0.26 ± 0.04	0.12 ± 0.04	0.0004	0.392
Cecum (full)	0.27 ± 0.13	0.38 ± 0.08	0.219	0.41 ± 0.15	0.25 ± 0.12	0.066	0.075
Feces	0.24 ± 0.19	0.46 ± 0.33	0.201	0.15 ± 0.27	0.06 ± 0.05	0.444	0.017
Heart	0.78 ± 0.16	1.09 ± 0.40	0.099	4.02 ± 1.84	1.90 ± 1.12	0.045	0.186
Kidneys	3.56 ± 0.85	6.36 ± 1.72	0.006	4.80 ± 1.18	7.09 ± 5.92	0.441	0.803
Large intestine (empty)	0.52 ± 0.11	0.92 ± 0.18	0.005	1.27 ± 0.74	1.51 ± 1.87	0.803	0.524
Liver	9.22 ± 0.69	13.74 ± 3.00	0.006	51.88 ± 9.62	81.32 ± 33.15	0.173	0.026
Lungs	1.28 ± 0.18	2.63 ± 0.87	0.006	11.62 ± 8.24	3.93 ± 3.37	0.076	0.442
Lymph nodes	0.72 ± 0.14	1.22 ± 0.34	0.009	3.21 ± 2.06	5.11 ± 9.17	0.710	0.459
Muscle	0.19 ± 0.07	0.27 ± 0.08	0.104	0.19 ± 0.03	0.15 ± 0.12	0.429	0.073
Ovaries	2.15 ± 0.35	4.53 ± 1.30	0.002	6.39 ± 3.26	8.67 ± 9.23	0.624	0.375
Pancreas	0.31 ± 0.06	0.56 ± 0.20	0.016	0.65 ± 0.15	0.51 ± 0.30	0.361	0.746
Plasma/whole blood ratio	1.67 ± 0.06	1.82 ± 0.22	0.128	2.45 ± 0.63	1.95 ± 0.32	0.136	0.487
Salivary glands	0.33 ± 0.04	0.62 ± 0.30	0.040	1.04 ± 0.55	0.71 ± 0.61	0.384	0.771
Skin	0.78 ± 0.16	1.32 ± 0.27	0.003	1.16 ± 0.42	2.08 ± 4.10	0.640	0.695
Small intestine (empty)	2.39 ± 1.60	3.51 ± 1.32	0.284	8.34 ± 4.06	4.38 ± 5.08	0.198	0.725
Spleen	4.07 ± 0.61	7.63 ± 1.39	0.0003	38.19 ± 3.79	49.33 ± 19.89	0.281	0.009
Stomach (full)	0.59 ± 0.08	0.73 ± 0.24	0.175	3.14 ± 1.29	2.69 ± 2.59	0.733	0.168
Thyroid glands	1.17 ± 0.36	2.06 ± 2.91	0.455	2.13 ± 0.79	2.10 ± 2.28	0.984	0.984
Tumor	0.91 ± 0.13	1.22 ± 0.38	0.085	1.93 ± 0.53	0.89 ± 0.74	0.034	0.394
Urinary bladder (empty)	7.06 ± 7.23	5.10 ± 6.41	0.668	10.76 ± 8.25	3.92 ± 4.93	0.126	0.725
Urine	482.64 ± 229.30	408.30 ± 103.21	0.575	10.54 ± 1.74	14.59 ± 2.40	0.076	0.0001
Uterus	1.98 ± 0.84	3.66 ± 1.18	0.025	4.28 ± 4.62	1.94 ± 1.94	0.298	0.128
White adipose tissue	0.60 ± 0.42	0.52 ± 0.34	0.762	0.59 ± 0.39	0.46 ± 0.40	0.573	0.757

a **[**^**18**^**F]MSNA-PO** and **[**^**18**^**F]MSNA-PS** data are reproduced from previously published paper.^44^ %ID/g = percentage of injected radioactivity dose per gram of tissue.

b [^18^F]Fol-MSNA-PO vs [^18^F]Fol-MSNA-PS.

**Figure 5:**
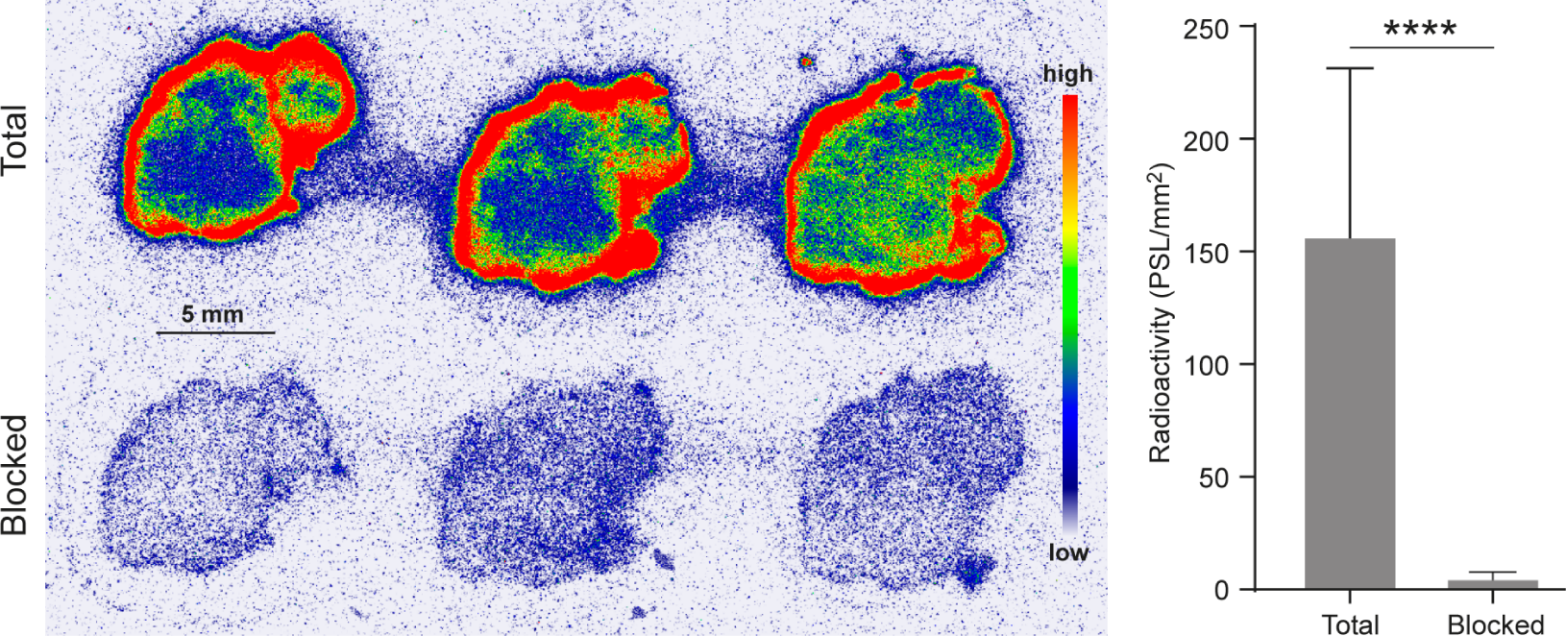
*In vitro* binding of **[**^**18**^**F]FOL,** a known tracer that binds to folate receptors, to HCC1954 tumor cryosections. A molar excess of an unlabeled **Fol-[TCO]MSNA-PO** blocking agent led to a significant reduction in tracer binding, indicating that **Fol-[TCO]MSNA-PO** binds specifically to folate receptors. PSL/mm^2^ = photostimulated luminescence per square millimeter. ****p < 0.0001.

Binding of **[**^**18**^**F]FOL** on tissue cryosections likewise confirmed the presence of FR-positive cells in HCC1954 tumors. Preincubation with excess unlabeled **Fol-[TCO]MSNA-PO** resulted in a 96.5 ± 0.7% reduction in **[**^**18**^**F]FOL** binding, confirming that **Fol-[TCO]MSNA-PO** binds to FRs (Figure 5).

## 4 Discussion

Recently, MSNAs have gained much attention as potential vehicles for the delivery of therapeutical ONs to target sites. Therefore, modifications to MSNA to ensure efficient delivery to target sites or tissues are a crucial area of investigation. Here, we radiolabeled folate-decorated MSNAs and examined the impact of the folate decoration on biodistribution.

Folate is an attractive targeting ligand due to its small size and high affinity for FRs, which are expressed at high levels by many tumors.^51^ Several methods are used to incorporate folate into nanostructures, including covalent conjugation of ONs to folate,^52^ hybridization-mediated assembly into folate-containing costructs,^53^ and construction of folate-functionalized liposomes.^30,54^ In our case, hybridization-mediated structures were used to affect the biodistribution/targeting properties of PO/PS-MSNA, recently studied in our group.^44^

Various studies have explored the use of folate moieties as functional decorations for targeted ON delivery.^28−32,54−56^ For instance, folate conjugation facilitates delivery of small interfering RNAs (siRNAs) to KB/GFPLuc tumor cells.^32^ Successful cell-based studies were also conducted by Leamon et al., who observed delivery of folate-targeted liposome-encapsulated ONs into FR-bearing cells; however, *in vivo* studies did not demonstrate tumor delivery.^54^ More promising *in vivo* results were obtained using DNA/siRNA tetrahedral nanoparticles in tumor-bearing mice, which demonstrated accumulation in the primary tumor and prolonged circulation in the blood.^28^

The IEDDA reaction is a valuable tool in the field of radiopharmaceutical chemistry due to its selective nature, rapid reaction kinetics, and occurrence at physiological temperatures. These characteristics are essential when working with short-lived radionuclides such as ^18^F.^43^ In our study, the IEDDA reaction between **[**^**18**^**F]FDG-Tz** and folate-decorated MSNAs resulted in high radiochemical conversion (82.7 ± 15.7%), as determined by radio-TLC, as well as an excellent RCP of >99%. These results align with those of our previous studies in which we radiolabeled a TCO-functionalized MSNA lacking a folate moiety; in that study, the IEDDA reaction also yielded an RCY >80%,^44^ indicating that folate decoration does not affect the efficiency of MSNA radiolabeling; however, the radiolabeling efficiency was influenced by the Tz-to-TCO ratio, necessitating an excess of TCO to achieve close to a 100% conversion in the reaction.

Herein, we found that folate led to increased uptake of MSNAs (one with a native PO backbone and one with a PS backbone) by the liver, kidneys, and spleen with increased tumor-to-muscle ratios. Considering the high expression of FRs in organs such as the kidneys,^57^ liver, and spleen,^58,59^ these changes in biodistribution could be attributed to FR binding. Taken together, these findings align with previously reported increases in uptake of DNA/siRNA tetrahedral nanoparticles by the kidneys.^28^

The tumor-to-muscle ratio was highest after the injection of **[**^**18**^**F]Fol-MSNA-PS**, exhibiting an increasing trend toward the end of the 60 min scan (Figure 4); however, the difference was not statistically significant due to data variations. Notably, folate decoration of **[**^**18**^**F]Fol-MSNA-PS** led to faster blood clearance than that of **[**^**18**^**F]MSNA-PS**, which resulted in a better tumor-to-muscle ratio.

The HCC1954 cells used in this study are known to be HER2-positive and express FR-α,^60,61^ and we confirmed this ourselves with flow cytometry. To confirm the FR-binding nature of the folate-decorated MSNAs, we conducted *in vitro* analyses utilizing a tracer that binds to FRs, **[**^**18**^**F]FOL**,^49^ along with a molar excess of **Fol-[TCO]MSNA-PO** as a blocking agent. **[**^**18**^**F]FOL** binding verified the expression of FRs in HCC1954 tumors, and the ability of **Fol-[TCO]MSNA-PO** to block **[**^**18**^**F]FOL** binding confirms that it binds to FRs.

We recognize that our study has some limitations. The modest increase in tumor uptake after administration of folate-decorated MSNAs (12 equiv folate/MSNA) compared with nonfolate MSNAs may be attributed to the presence of unlabeled Fol-[TCO]MSNAs in the final injection solution. Unlabeled Fol-[TCO]MSNAs originate from excess TCO-functionalized Fol-[TCO]MSNAs used during the IEDDA reaction, which were not removed during the purification step. The homogeneity and authenticity of radiolabeled MSNAs were verified by PAGE (Figure S4), radio-TLC, radio-SEC (Figure S7), and SEC-MALS (Figures S8 and S9) in the formulation solution. Enzymatic stability of the MSNAs was evaluated in the presence of DNase I *in vitro*, but their stability was not studied *in vivo*. According to the *in vitro*-stability tests with DNase I, **[TCO]MSNA-PO** and its hybridization complex **Fol-[TCO]MSNA-PO** were prone to rapid degradation, which may confound the interpretability of the biodistribution results. The functionality of **Fol-[TCO]MSNA-PS** in the presence of DNase I demonstrated that PS-backbone-stabilized MSNAs, decorated with hybridization-mediated Fol-ligands, undergo enzymatic cleavage, releasing the active intact cargo (**[TCO]MSNA-PS**). Targeting specificity was addressed only by *in vitro* autoradiography experiments. Our results also indicate that the pharmacokinetics of the structures significantly affect tumor uptake. *In vitro* cellular uptake and activity experiments to downregulate HER2 would not reliably predict the *in vivo* behavior of the MSNAs, and therefore, these studies were excluded. We attempted to investigate stability of the MSNAs in plasma, but faced challenges due to plasma protein binding. This was unfortunate as detailed stability kinetics of MSNAs *in vivo* would have explained part of the biodistribution and tumor-targeting properties of the MSNAs. In addition, the biocompatibility of the synthesized compounds, particularly their cytotoxic effects on normal cells, remains to be evaluated.

While this study focuses on the targeting and uptake characteristics of folate-decorated MSNAs, the potential for recurrence or resurgence of malignancy following therapeutic intervention remains an important challenge. Future investigations should assess the long-term efficacy of these systems in mitigating cancer relapse, particularly in the context of FR-expressing tumors.

## 5 Conclusions

In this study, we investigated the ability of folate-decorated 12-armed [60]fullerene-based MSNAs to target FR-expressing HCC1954^60^ tumors in female mice. Two MSNAs, one with a native phosphodiester backbone and one with a phosphorothioate backbone, were modified with folate and then used in experiments alongside previously published nonfolate structures. For the purpose of biological evaluation, MSNAs were site-specifically labeled with **[**^**18**^**F]FDG-Tz** using the fast and efficient IEDDA click chemistry approach, and biodistribution in mice was studied using dynamic PET/CT. The presence of the folate moiety in MSNAs resulted in a higher tumor-to-muscle ratio, as well as increased kidney uptake, which suggests that the tracers bind to FRs. However, the increase was statistically significant for only structures with a PO backbone. *In vitro* blocking studies confirmed the specific binding of **Fol-[TCO]MSNA-PO** to FRs. The observed accumulation of **[**^**18**^**F]Fol-MSNA-PS** and **[**^**18**^**F]MSNA-PS** in the liver underscored the critical role of the backbone in determining the excretion route, with nanoparticles featuring native PO backbones being eliminated via nuclease-mediated degradation and urinary excretion. The data presented herein confirm the feasibility of decorating MSNAs with a folate moiety for targeting purposes, as well as the FR-binding nature of folate-decorated MSNAs; however, further investigations should consider structural improvements to enhance the tumor-targeting properties.

## Supporting Information Available

RP-HPLC and ESI-TOF MS characterization of **Fol**-**ON**; PAGE and SEC-MALS of MSNAs; *ex vivo* biodistribution and statistical analysis; autoradiography and hematoxylin-eosin (H&E) staining of 20 μm tumor slices (PDF)

## Abbreviations

%ID/g: percentage of injected radioactivity dose per gram of tissue
CT: computed tomography
DMSO: dimethyl sulfoxide
FR: folate receptor
HER2: human epidermal growth factor receptor 2
HPLC: high-performance liquid chromatography
IEDDA: inverse electron demand Diels–Alder
MALS: multiple angle light scattering
MSNA: molecular spherical nucleic acid
ON: oligonucleotide
PAGE: polyacrylamide gel electrophoresis
PBS: phosphate-buffered saline
PET: positron emission tomography
PO: phosphodiester
PS: phosphorothioate
ROI: region of interest
SEC: size-exclusion chromatography
siRNA: small interfering RNA
SNA: spherical nucleic acid
SUV: standardized uptake value
TBE: tris-borate-EDTA
TCO: *trans-*cyclooctene
TLC: thin-layer chromatography
Tz: tetrazine

## References

[ref1] Barnaby S. N.; Sita T. L.; Petrosko S. H.; Stegh A. H.; Mirkin C. A. Therapeutic Applications of Spherical Nucleic Acids. Cancer Treat. Res. 2015, 166, 23–50. 10.1007/978-3-319-16555-4_2. 25895863

[ref2] Li H.; Zhang B.; Lu X.; Tan X.; Jia F.; Xiao Y.; Cheng Z.; Li Y.; Silva D. O.; Schrekker H. S.; Zhang K.; Mirkin C. A. Molecular Spherical Nucleic Acids. Proc. Natl. Acad. Sci. U. S. A. 2018, 115 (17), 4340–4344. 10.1073/pnas.1801836115. 29632214 PMC5924931

[ref3] Banga R. J.; Chernyak N.; Narayan S. P.; Nguyen S. T.; Mirkin C. A. Liposomal Spherical Nucleic Acids. J. Am. Chem. Soc. 2014, 136 (28), 9866–9869. 10.1021/ja504845f. 24983505 PMC4280063

[ref4] Brodin J. D.; Sprangers A. J.; McMillan J. R.; Mirkin C. A. DNA-Mediated Cellular Delivery of Functional Enzymes. J. Am. Chem. Soc 2015, 137 (47), 14838–14841. 10.1021/jacs.5b09711. 26587747 PMC5831182

[ref5] Juliano R.; Bauman J.; Kang H.; Ming X. Biological Barriers to Therapy with Antisense and SiRNA Oligonucleotides. Mol. Pharmaceutics 2009, 6 (3), 686–695. 10.1021/mp900093r. PMC2758224 19397332

[ref6] de Wolf H. K.; Snel C. J.; Verbaan F. J.; Schiffelers R. M.; Hennink W. E.; Storm G. Effect of Cationic Carriers on the Pharmacokinetics and Tumor Localization of Nucleic Acids after Intravenous Administration. Int. J. Pharm. 2007, 331, 167–175. 10.1016/j.ijpharm.2006.10.029. 17134859

[ref7] Lebedeva I.; Stein C. A. Antisense Oligonucleotides: Promise and Reality. Annu. Rev. Pharmacol. Toxicol. 2001, 41, 403–419. 10.1146/annurev.pharmtox.41.1.403. 11264463

[ref8] Choi C. H. J.; Hao L.; Narayan S. P.; Auyeung E.; Mirkin C. A. Mechanism for the Endocytosis of Spherical Nucleic Acid Nanoparticle Conjugates. Proc. Natl. Acad. Sci. U. S. A. 2013, 110 (19), 7625–7630. 10.1073/pnas.1305804110. 23613589 PMC3651452

[ref9] Patel P. C.; Giljohann D. A.; Daniel W. L.; Zheng D.; Prigodich A. E.; Mirkin C. A. Scavenger Receptors Mediate Cellular Uptake of Polyvalent Oligonucleotide-Functionalized Gold Nanoparticles. Bioconjugate Chem. 2010, 21 (12), 2250–2256. 10.1021/bc1002423. PMC3241523 21070003

[ref10] Massich M. D.; Giljohann D. A.; Seferos D. S.; Ludlow L. E.; Horvath C. M.; Mirkin C. A. Regulating Immune Response Using Polyvalent Nucleic Acid-Gold Nanoparticle Conjugates. Mol. Pharm. 2009, 6 (6), 1934–1940. 10.1021/mp900172m. 19810673 PMC3241524

[ref11] Seferos D. S.; Prigodich A. E.; Giljohann D. A.; Patel P. C.; Mirkin C. A. Polyvalent DNA Nanoparticle Conjugates Stabilize Nucleic Acids. Nano Lett. 2009, 9 (1), 308–311. 10.1021/nl802958f. 19099465 PMC3918421

[ref12] Barnaby S. N.; Perelman G. A.; Kohlstedt K. L.; Chinen A. B.; Schatz G. C.; Mirkin C. A. Design Considerations for RNA Spherical Nucleic Acids (SNAs). Bioconjugate Chem. 2016, 27 (9), 2124–2131. 10.1021/acs.bioconjchem.6b00350. PMC5034328 27523252

[ref13] Bousmail D.; Amrein L.; Fakhoury J. J.; Fakih H. H.; Hsu J. C. C.; Panasci L.; Sleiman H. F. Precision Spherical Nucleic Acids for Delivery of Anticancer Drugs. Chem. Sci. 2017, 8 (9), 6218–6229. 10.1039/C7SC01619K. 28989655 PMC5628336

[ref14] Chinen A. B.; Guan C. M.; Ko C. H.; Mirkin C. A. The Impact of Protein Corona Formation on the Macrophage Cellular Uptake and Biodistribution of Spherical Nucleic Acids. Small 2017, 13 (16), 1603847 10.1002/smll.201603847. PMC5493144 28196309

[ref15] Tähtinen V.; Gulumkar V.; Maity S. K.; Yliperttula A. M.; Siekkinen S.; Laine T.; Lisitsyna E.; Haapalehto I.; Viitala T.; Vuorimaa-Laukkanen E.; Yliperttula M.; Virta P. Assembly of Bleomycin Saccharide-Decorated Spherical Nucleic Acids. Bioconjugate Chem. 2022, 33 (1), 206–218. 10.1021/acs.bioconjchem.1c00539. PMC8778632 34985282

[ref16] Zhang K.; Hao L.; Hurst S. J.; Mirkin C. A. Antibody-Linked Spherical Nucleic Acids for Cellular Targeting. J. Am. Chem. Soc. 2012, 134 (40), 16488–16491. 10.1021/ja306854d. 23020598 PMC3501255

[ref17] Gulumkar V.; Äärelä A.; Moisio O.; Rahkila J.; Tähtinen V.; Leimu L.; Korsoff N.; Korhonen H.; Poijärvi-Virta P.; Mikkola S.; Nesati V.; Vuorimaa-Laukkanen E.; Viitala T.; Yliperttula M.; Roivainen A.; Virta P. Controlled Monofunctionalization of Molecular Spherical Nucleic Acids on a Buckminster Fullerene Core. Bioconjugate Chem. 2021, 32 (6), 1130–1138. 10.1021/acs.bioconjchem.1c00187. PMC8382215 33998229

[ref18] Distler M. E.; Teplensky M. H.; Bujold K. E.; Kusmierz C. D.; Evangelopoulos M.; Mirkin C. A. DNA Dendrons as Agents for Intracellular Delivery. J. Am. Chem. Soc. 2021, 143 (34), 13513–13518. 10.1021/jacs.1c07240. 34410116 PMC8582297

[ref19] Äärelä A.; Räsänen K.; Holm P.; Salo H.; Virta P. Synthesis of Site-Specific Antibody-[60]Fullerene-Oligonucleotide Conjugates for Cellular Targeting. ACS Appl. Bio Mater. 2023, 6 (8), 3189–3198. 10.1021/acsabm.3c00318. PMC10445261 37432881

[ref20] Zheng Y.; Cantley L. C. Toward a Better Understanding of Folate Metabolism in Health and Disease. J. Exp. Med. 2019, 216 (2), 253–266. 10.1084/jem.20181965. 30587505 PMC6363433

[ref21] Locasale J. W. S. Serine, glycine and one-carbon units: cancer metabolism in full circle. Nat. Rev. Cancer 2013, 13 (8), 572–583. 10.1038/nrc3557. 23822983 PMC3806315

[ref22] Chen C.; Ke J.; Edward Zhou X.; Yi W.; Brunzelle J. S.; Li J.; Yong E. L.; Xu H. E.; Melcher K. Structural Basis for Molecular Recognition of Folic Acid by Folate Receptors. Nature 2013, 500 (7463), 486–489. 10.1038/nature12327. 23851396 PMC5797940

[ref23] Toffoli G.; Cernigoi C.; Russo A.; Gallo A.; Bagnoli M.; Boiocchi M. Overexpression of Folate Binding Protein in Ovarian Cancers. Int. J. Cancer 1997, 74 (2), 193–198. 10.1002/(SICI)1097-0215(19970422)74:2<193::AID-IJC10>3.0.CO;2-F. 9133455

[ref24] Cheung A.; Bax H. J.; Josephs D. H.; Ilieva K. M.; Pellizzari G.; Opzoomer J.; Bloomfield J.; Fittall M.; Grigoriadis A.; Figini M.; Canevari S.; Spicer J. F.; Tutt A. N.; Karagiannis S. N. Targeting Folate Receptor Alpha for Cancer Treatment. Oncotarget 2016, 7 (32), 52553–52574. 10.18632/oncotarget.9651. 27248175 PMC5239573

[ref25] Varaganti P.; Buddolla V.; Lakshmi B. A.; Kim Y. J. Recent Advances in Using Folate Receptor 1 (FOLR1) for Cancer Diagnosis and Treatment, with an Emphasis on Cancers That Affect Women. Life Sci. 2023, 326, 121802 10.1016/j.lfs.2023.121802. 37244363

[ref26] Farran B.; Montenegro R. C.; Kasa P.; Pavitra E.; Huh Y. S.; Han Y. K.; Kamal M. A.; Nagaraju G. P.; Rama Raju G. S. Folate-Conjugated Nanovehicles: Strategies for Cancer Therapy. Mater. Sci. Eng., C 2020, 107, 110341 10.1016/j.msec.2019.110341. 31761235

[ref27] Sudimack J.; Lee R. J. Targeted Drug Delivery via the Folate Receptor. Adv. Drug Delivery Rev. 2000, 41 (2), 147–162. 10.1016/S0169-409X(99)00062-9. 10699311

[ref28] Lee H.; Lytton-Jean A. K. R.; Chen Y.; Love K. T.; Park A. I.; Karagiannis E. D.; Sehgal A.; Querbes W.; Zurenko C. S.; Jayaraman M.; Peng C. G.; Charisse K.; Borodovsky A.; Manoharan M.; Donahoe J. S.; Truelove J.; Nahrendorf M.; Langer R.; Anderson D. G. Molecularly Self-Assembled Nucleic Acid Nanoparticles for Targeted in Vivo SiRNA Delivery. Nat. Nanotechnol. 2012, 7 (6), 389–393. 10.1038/nnano.2012.73. 22659608 PMC3898745

[ref29] Kabilova T. O.; Shmendel E. V.; Gladkikh D. V.; Chernolovskaya E. L.; Markov O. V.; Morozova N. G.; Maslov M. A.; Zenkova M. A. Targeted Delivery of Nucleic Acids into Xenograft Tumors Mediated by Novel Folate-Equipped Liposomes. Eur. J. Pharm. Biopharm. 2018, 123, 59–70. 10.1016/j.ejpb.2017.11.010. 29162508

[ref30] Gabizon A.; Horowitz A. T.; Goren D.; Tzemach D.; Shmeeda H.; Zalipsky S. In Vivo Fate of Folate-Targeted Polyethylene-Glycol Liposomes in Tumor-Bearing Mice. Clin. Cancer Res. 2003, 9 (17), 6551–6559. 14695160

[ref31] Salim L.; Islam G.; Desaulniers J. P. Targeted Delivery and Enhanced Gene-Silencing Activity of Centrally Modified Folic Acid-SiRNA Conjugates. Nucleic Acids Res. 2020, 48 (1), 75–85. 10.1093/nar/gkz1115. 31777918 PMC6943128

[ref32] Dohmen C.; Fröhlich T.; Lächelt U.; Röhl I.; Vornlocher H. P.; Hadwiger P.; Wagner E. Defined Folate-PEG-SiRNA Conjugates for Receptor-Specific Gene Silencing. Mol. Ther. Nucleic Acids 2012, 1 (1), e7 10.1038/mtna.2011.10. 23344624 PMC3381594

[ref33] Shmendel E. V.; Puchkov P. A.; Maslov M. A. Design of Folate-Containing Liposomal Nucleic Acid Delivery Systems for Antitumor Therapy. Pharmaceutics 2023, 15 (5), 1400 10.3390/pharmaceutics15051400. 37242642 PMC10221727

[ref34] Unida V.; Vindigni G.; Raniolo S.; Stolfi C.; Desideri A.; Biocca S. Folate-Functionalization Enhances Cytotoxicity of Multivalent DNA Nanocages on Triple-Negative Breast Cancer Cells. Pharmaceutics 2022, 14 (12), 2610 10.3390/pharmaceutics14122610. 36559104 PMC9786333

[ref35] Bogani G.; Coleman R. L.; Vergote I.; van Gorp T.; Ray-Coquard I.; Oaknin A.; Matulonis U.; O’Malley D.; Raspagliesi F.; Scambia G.; Monk B. J. Mirvetuximab Soravtansine-Gynx: First Antibody/Antigen-Drug Conjugate (ADC) in Advanced or Recurrent Ovarian Cancer. Int. J. Gynecol. Cancer 2024, 34 (4), 469–477. 10.1136/ijgc-2023-004924. 38101816

[ref36] Choi Y.; Baker J. R. Targeting Cancer Cells with DNA-Assembled Dendrimers: A Mix and Match Strategy for Cancer. Cell Cycle 2005, 4, 669–671. 10.4161/cc.4.5.1684. 15846063

[ref37] Agrahari V. The Exciting Potential of Nanotherapy in Brain-Tumor Targeted Drug Delivery Approaches. Neural Regen Res. 2017, 12 (2), 197–200. 10.4103/1673-5374.200796. 28400793 PMC5361495

[ref38] Kolb H. C.; Finn M. G.; Sharpless K. B. Click Chemistry: Diverse Chemical Function from a Few Good Reactions. Angew. Chem., Int. Ed. 2001, 40 (11), 2004–2021. 10.1002/1521-3773(20010601)40:11<2004::AID-ANIE2004>3.0.CO;2-5. 11433435

[ref39] Stéen E. J. L.; Jørgensen J. T.; Denk C.; Battisti U. M.; Nørregaard K.; Edem P. E.; Bratteby K.; Shalgunov V.; Wilkovitsch M.; Svatunek D.; Poulie C. B. M.; Hvass L.; Simón M.; Wanek T.; Rossin R.; Robillard M.; Kristensen J. L.; Mikula H.; Kjaer A.; Herth M. M. Lipophilicity and Click Reactivity Determine the Performance of Bioorthogonal Tetrazine Tools in Pretargeted in Vivo Chemistry. ACS Pharmacol. Transl. Sci. 2021, 4 (2), 824–833. 10.1021/acsptsci.1c00007. 33860205 PMC8033778

[ref40] Wang M.; Svatunek D.; Rohlfing K.; Liu Y.; Wang H.; Giglio B.; Yuan H.; Wu Z.; Li Z.; Fox J. Conformationally Strained Trans-Cyclooctene (STCO) Enables the Rapid Construction of ^18^F-PET Probes via Tetrazine Ligation. Theranostics 2016, 6 (6), 887–895. 10.7150/thno.14742. 27162558 PMC4860896

[ref41] Otaru S.; Paulus A.; Imlimthan S.; Kuurne I.; Virtanen H.; Liljenbäck H.; Tolvanen T.; Auchynnikava T.; Roivainen A.; Helariutta K.; Sarparanta M.; Airaksinen A. J. Development of [^18^F]AmBF3 Tetrazine for Radiolabeling of Peptides: Preclinical Evaluation and PET Imaging of [^18^F]AmBF3-PEG7-Tyr3-Octreotide in an AR42J Pancreatic Carcinoma Model. Bioconjugate Chem. 2022, 33 (7), 1393–1404. 10.1021/acs.bioconjchem.2c00231. PMC9305971 35709482

[ref42] Syvänen S.; Fang X. T.; Faresjö R.; Rokka J.; Lannfelt L.; Olberg D. E.; Eriksson J.; Sehlin D. Fluorine-18-Labeled Antibody Ligands for PET Imaging of Amyloid-β in Brain. ACS Chem. Neurosc.i 2020, 11 (24), 4460–4468. 10.1021/acschemneuro.0c00652. PMC7747219 33236886

[ref43] Handula M.; Chen K.-T.; Seimbille Y. IEDDA: An Attractive Bioorthogonal Reaction for Biomedical Applications. Molecules 2021, 26 (15), 4640 10.3390/molecules26154640. 34361793 PMC8347371

[ref44] Äärelä A.; Auchynnikava T.; Moisio O.; Liljenbäck H.; Andriana P.; Iqbal I.; Lehtimäki J.; Rajander J.; Salo H.; Roivainen A.; Airaksinen A. J.; Virta P. In Vivo Imaging of [60]Fullerene-Based Molecular Spherical Nucleic Acids by Positron Emission Tomography. Mol. Pharm. 2023, 20 (10), 5043–5051. 10.1021/acs.molpharmaceut.3c00370. 37531591 PMC10548468

[ref45] Kyriazi M. E.; El-Sagheer A. H.; Medintz I. L.; Brown T.; Kanaras A. G. An Investigation into the Resistance of Spherical Nucleic Acids against DNA Enzymatic Degradation. Bioconjugate Chem. 2022, 33 (1), 219–225. 10.1021/acs.bioconjchem.1c00540. 35001632

[ref46] Geary R. S.; Norris D.; Yu R.; Bennett C. F. Pharmacokinetics, Biodistribution and Cell Uptake of Antisense Oligonucleotides. Adv. Drug Delivery Rev. 2015, 87, 46–51. 10.1016/j.addr.2015.01.008. 25666165

[ref47] Auchynnikava T.; Äärelä A.; Liljenbäck H.; Järvinen J.; Andriana P.; Kovacs L.; Rautio J.; Rajander J.; Virta P.; Roivainen A.; Li X. G.; Airaksinen A. J. Tetrazine Glycoconjugate for Pretargeted Positron Emission Tomography Imaging of Trans-Cyclooctene-Functionalized Molecular Spherical Nucleic Acids. ACS Omega 2023, 8 (48), 45326–45336. 10.1021/acsomega.3c04041. 38075748 PMC10702189

[ref48] Elo P.; Li X. G.; Liljenbäck H.; Helin S.; Teuho J.; Koskensalo K.; Saunavaara V.; Marjamäki P.; Oikonen V.; Virta J.; Chen Q.; Low P. S.; Knuuti J.; Jalkanen S.; Airas L.; Roivainen A. Folate Receptor-Targeted Positron Emission Tomography of Experimental Autoimmune Encephalomyelitis in Rats. J. Neuroinflam. 2019, 16 (1), 252 10.1186/s12974-019-1612-3. PMC6892159 31796042

[ref49] Silvola J. M. U.; Li X. G.; Virta J.; Marjamäki P.; Liljenbäck H.; Hytönen J. P.; Tarkia M.; Saunavaara V.; Hurme S.; Palani S.; Hakovirta H.; Ylä-Herttuala S.; Saukko P.; Chen Q.; Low P. S.; Knuuti J.; Saraste A.; Roivainen A. Aluminum Fluoride-18 Labeled Folate Enables in Vivo Detection of Atherosclerotic Plaque Inflammation by Positron Emission Tomography. Sci. Rep. 2018, 8 (1), 9720 10.1038/s41598-018-27618-4. 29946129 PMC6018703

[ref50] Keinänen O.; Partelová D.; Alanen O.; Antopolsky M.; Sarparanta M.; Airaksinen A. J. Efficient Cartridge Purification for Producing High Molar Activity ^18^F-Glycoconjugates via Oxime Formation. Nucl. Med. Biol. 2018, 67, 27–35. 10.1016/j.nucmedbio.2018.10.001. 30380464

[ref51] Xia W.; Low P. S. Folate-Targeted Therapies for Cancer. J. Med. Chem. 2010, 53 (19), 6811–6824. 10.1021/jm100509v. 20666486

[ref52] Zhang K.; Wang Q.; Xie Y.; Mor G.; Sega E.; Low P. S.; Huang Y. Receptor-Mediated Delivery of SiRNAs by Tethered Nucleic Acid Base-Paired Interactions. Rna 2008, 14 (3), 577–583. 10.1261/rna.739308. 18218703 PMC2248269

[ref53] Thomas M.; Kularatne S. A.; Qi L.; Kleindl P.; Leamon C. P.; Hansen M. J.; Low P. S. Ligand-Targeted Delivery of Small Interfering RNAs to Malignant Cells and Tissues. Ann. N. Y. Acad. Sci. 2009, 1175 (1), 32–39. 10.1111/j.1749-6632.2009.04977.x. 19796075

[ref54] Leamon C. P.; Cooper S. R.; Hardee G. E. Folate-Liposome-Mediated Antisense Oligodeoxynucleotide Targeting to Cancer Cells: Evaluation in Vitro and in Vivo. Bioconjugate Chem. 2003, 14 (4), 738–747. 10.1021/bc020089t. 12862426

[ref55] Chiu S.-J.; Marcucci G.; Lee R. J. Efficient Delivery of an Antisense Oligodeoxyribonucleotide Formulated in Folate Receptor-Targeted Liposomes. Anticancer Res. 2006, 26 (2A), 1049–1056. 16619505

[ref56] Zhou W.; Yuan X.; Wilson A.; Yang L.; Mokotoff M.; Pitt B.; Li S. Efficient Intracellular Delivery of Oligonucleotides Formulated in Folate Receptor-Targeted Lipid Vesicles. Bioconjugate Chem. 2002, 13 (6), 1220–1225. 10.1021/bc025569z. 12440856

[ref57] Parker N.; Turk M. J.; Westrick E.; Lewis J. D.; Low P. S.; Leamon C. P. Folate Receptor Expression in Carcinomas and Normal Tissues Determined by a Quantitative Radioligand Binding Assay. Anal. Biochem. 2005, 338 (2), 284–293. 10.1016/j.ab.2004.12.026. 15745749

[ref58] Gnesin S.; Müller J.; Burger I. A.; Meisel A.; Siano M.; Früh M.; Choschzick M.; Müller C.; Schibli R.; Ametamey S. M.; Kaufmann P. A.; Treyer V.; Prior J. O.; Schaefer N. Radiation Dosimetry of ^18^F-AzaFol: A First in-Human Use of a Folate Receptor PET Tracer. EJNMMI Res. 2020, 10 (1), 32 10.1186/s13550-020-00624-2. 32270313 PMC7142191

[ref59] Boss S. D.; Ametamey S. M. Development of Folate Receptor–Targeted PET Radiopharmaceuticals for Tumor Imaging—A Bench-to-Bedside Journey. Cancers 2020, 12 (6), 1508 10.3390/cancers12061508. 32527010 PMC7352234

[ref60] Matsunaga Y.; Yamaoka T.; Ohba M.; Miura S.; Masuda H.; Sangai T.; Takimoto M.; Nakamura S.; Tsurutani J. Novel Anti-FOLR1 Antibody–Drug Conjugate MORAb-202 in Breast Cancer and Non-Small Cell Lung Cancer Cells. Antibodies 2021, 10 (1), 6 10.3390/antib10010006. 33535554 PMC7930947

[ref61] Cheng X.; Li J.; Tanaka K.; Majumder U.; Milinichik A. Z.; Verdi A. C.; Maddage C. J.; Rybinski K. A.; Fernando S.; Fernando D.; Kuc M.; Furuuchi K.; Fang F.; Uenaka T.; Grasso L.; Albone E. F. MORAb-202, an Antibody–Drug Conjugate Utilizing Humanized Anti-Human FRa Farletuzumab and the Microtubule-Targeting Agent Eribulin, Has Potent Antitumor Activity. Mol. Cancer Ther. 2018, 17 (12), 2665–2675. 10.1158/1535-7163.MCT-17-1215. 30262588

