# Supplementary material for: Biological Evaluation of Molecular Spherical Nucleic Acids: Targeting Tumors via a Hybridization-Based Folate Decoration
Source: ACS Omega. 2025 Feb 4;10(6):6003–14. doi: 10.1021/acsomega.4c10047 (PMC11840764; doi:10.1021/acsomega.4c10047)
Supplement: Supplementary file 1 — ao4c10047_si_001.pdf [file ao4c10047_si_001.pdf]

Supporting information to:

Biological evaluation of molecular spherical nucleic acids: targeting tumors via a hybridization-based folate decoration

Tatsiana Auchynnikava[a,b],[‡] Antti Äärelä[b,c],[‡] Olli Moisio[a], Heidi Liljenbäck[a,d], Putri Andriana[a], Imran Iqbal[a], Toni Laine[b], Senthil Palani[a], Jyrki Lehtimäki[c], Johan Rajander[e], Harri Salo[c], Anu J. Airaksinen[a,b], Pasi Virta[b] and Anne Roivainen[a,d,f]*

[a]Turku PET Centre, University of Turku and Turku University Hospital, FI-20520 Turku, Finland; [b]Department of Chemistry, University of Turku, FI-20500 Turku, Finland; [c]Research and Development, Orion Pharma, FI-20380 Turku, Finland; [d]Turku Center for Disease Modeling, University of Turku, FI-20520 Turku Finland; [e]Turku PET Centre, Accelerator Laboratory, Åbo Akademi University, FI-20520 Turku, Finland; [f]InFLAMES Research Flagship, University of Turku, FI-20520 Turku, Finland.

* Corresponding author: Anne Roivainen, email: anne.roivainen@utu.fi

PAGE analysis of the MSNAs

Native 6% Tris-borate-EDTA (TBE) acrylamide gels were used to analyze MSNAs. A 10 cm × 10 cm precast gel (Thermo Fisher Scientific, Waltham, MA, USA) was installed in a vertical electrophoresis apparatus, which was then filled with running buffer comprising 90 mM Tris, 90 mM borate, and 2 mM EDTA (pH 8.3). For analysis, MSNA samples (a mixture of 5 µL [taken from a 0.1 µM MSNA stock] and 5 µL of TBE sample buffer), along with a DNA ladder (100 to 1000 base pairs, used to verify the run's quality and consistency rather than the size of the MSNAs), were loaded onto the gel and electrophoresed at a steady 200 V for around 30 min. Following electrophoresis, the gel was stained with SYBR™ Gold Nucleic Acid Stain (Thermo Fisher Scientific, Waltham, MA, USA) and imaged using a Gel Doc imaging system (Bio-Rad, Hercules, CA, USA).

SEC-MALS experiments

SEC-MALS was performed using a 1260 Infinity II HPLC system (sampler, pump, and UV–vis detector; Agilent Technologies, Santa Barbara, CA, USA) equipped with a miniDAWN light scattering detector and an Optilab refractive index detector (Wyatt Technologies, Santa Barbara, CA, USA). The analysis was conducted over a 20 min period using an AdvanceBio SEC column (300Å 2.7 µm 4.6 × 300 mm; Agilent Technologies, Santa Clara, CA, USA), with a mobile phase of 150 mM sodium phosphate (pH 7.0) and a flow rate of 0.2 mL/min. For each analysis, 10 µL of a 1 mg/mL MSNA sample in Milli-Q water was introduced into the pre-equilibrated column. The molecular weight was determined using the refractive index, with an average refractive index increment (dn/dc) of 0.1703 mL/g.

Enzymatic stability of the MSNAs

The MSNAs (dissolved in PBS) were diluted into 10 mM tris-HCl (pH 7.5) with 2.5 mM $MgCl_2$ and 0.1 mM $CaCl_2$. The solutions (30 µL, c(MSNA) = 0.17 µM) were incubated at 37 °C with DNase I (1 U/nmol of effective oligonucleotide content). After 1 h and 5 h, 10 µL samples from the reactions were taken and added into 5 µL of TBE sample buffer on ice. 2 µL of each sample was loaded onto a 6% TBE acrylamide gel, which was electrophoresed at a constant 100 V for 50 min (Figure S5). After completion, the gel was stained by SYBRTM Gold Nucleic Acid Stain and imaged.

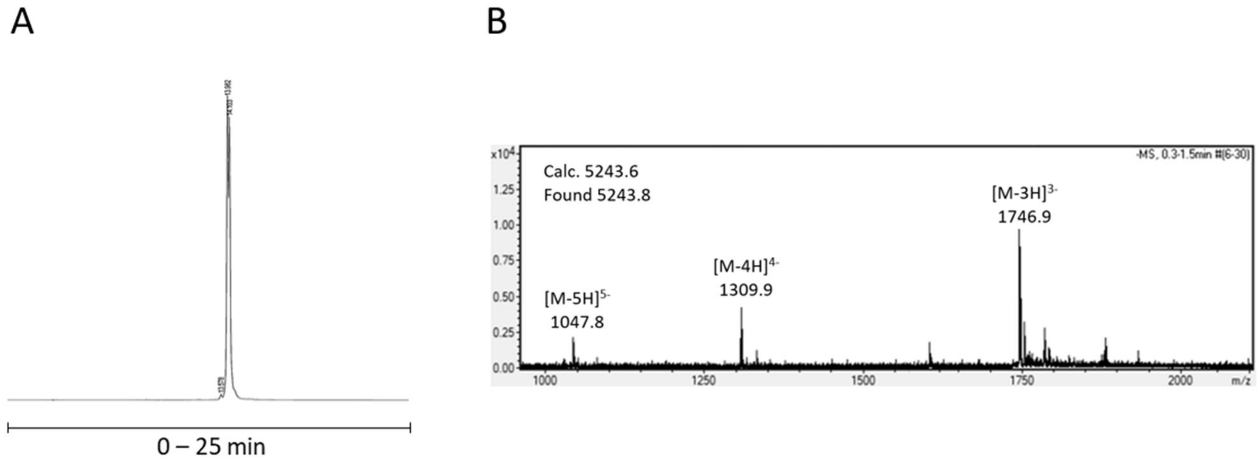

Figure S1. Characterization of the phosphodiester oligonucleotide Fol-ON moiety. A) RP-HPLC profile and B) MS (ESI-TOF) spectrum. RP-HPLC conditions: An analytical RP column (250 × 4.6 mm, 5 µm); detection at λ = 260 nm; gradient elution (0–25 min) from 5% to 45% MeCN in 50 mM triethylammonium acetate; flow rate, 1.0 mL min^{-1}.

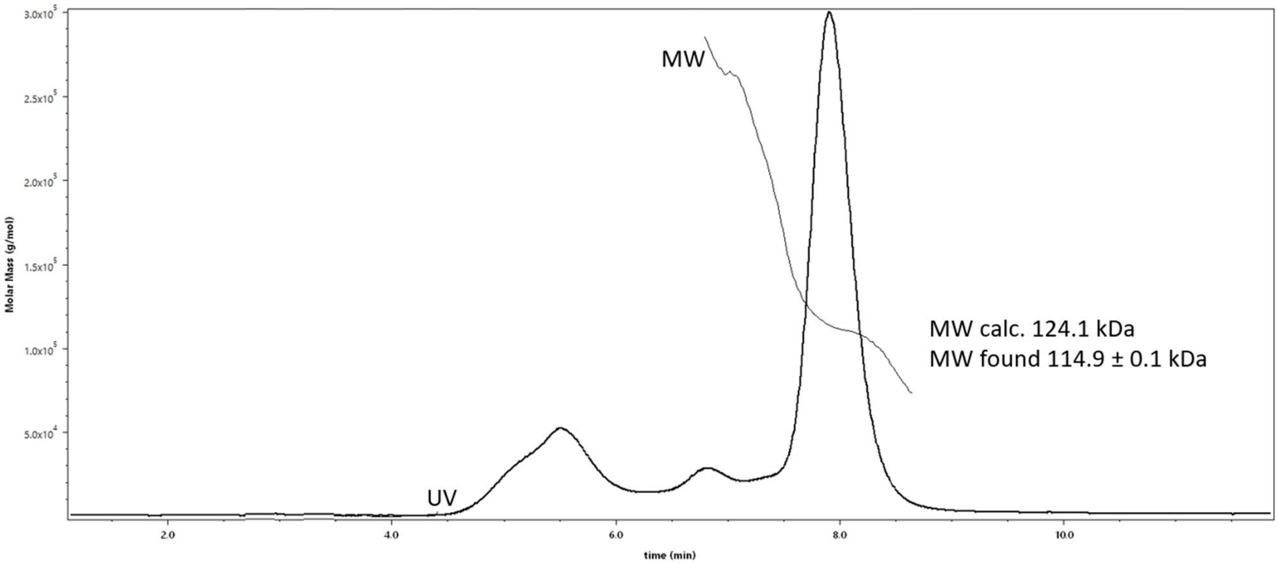

Figure S2. SEC-MALS profile of Fol-[TCO]MSNA-PO.

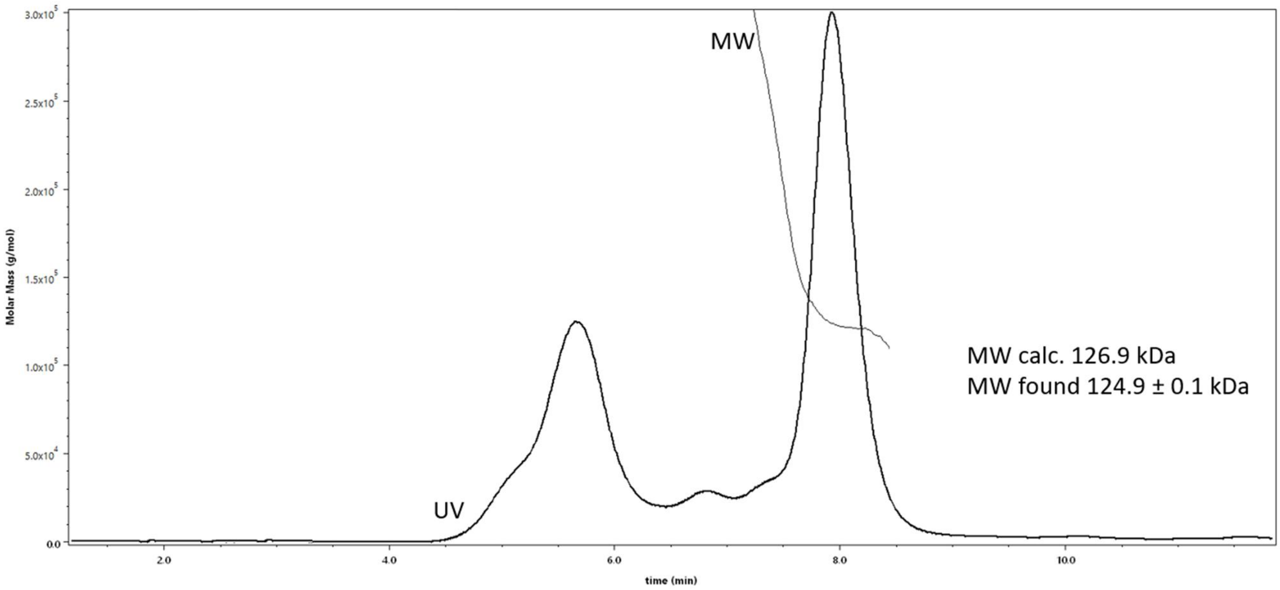

Figure S3. SEC-MALS profile of Fol-[TCO]MSNA-PS.

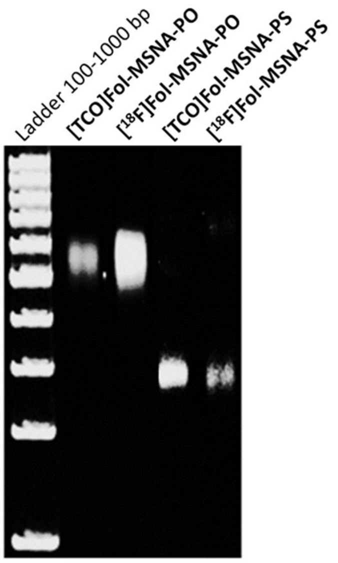

Figure S4. PAGE analysis of folate-decorated MSNAs after radiolabeling.

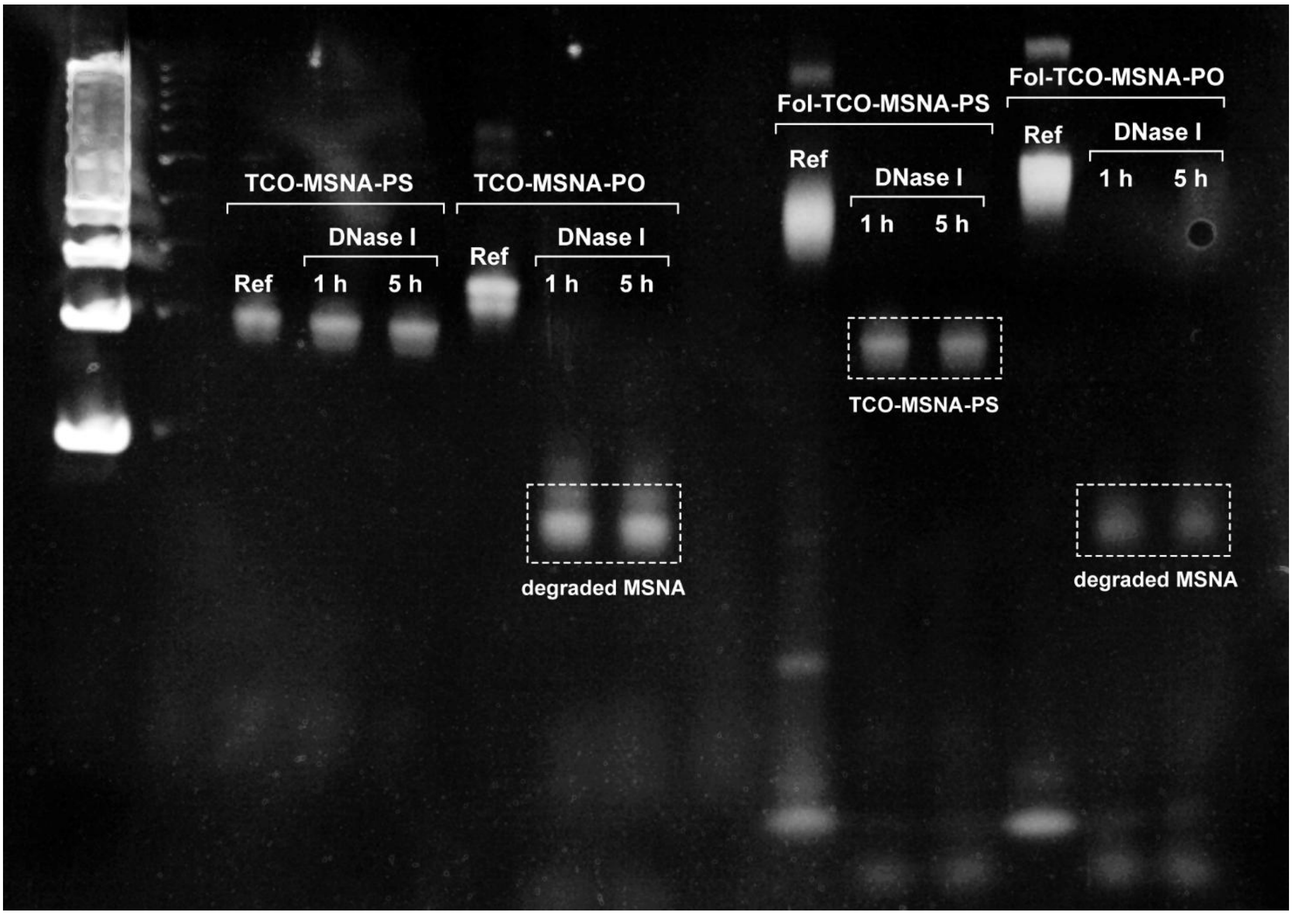

Figure S5. Enzymatic stability tests of the MSNAs.

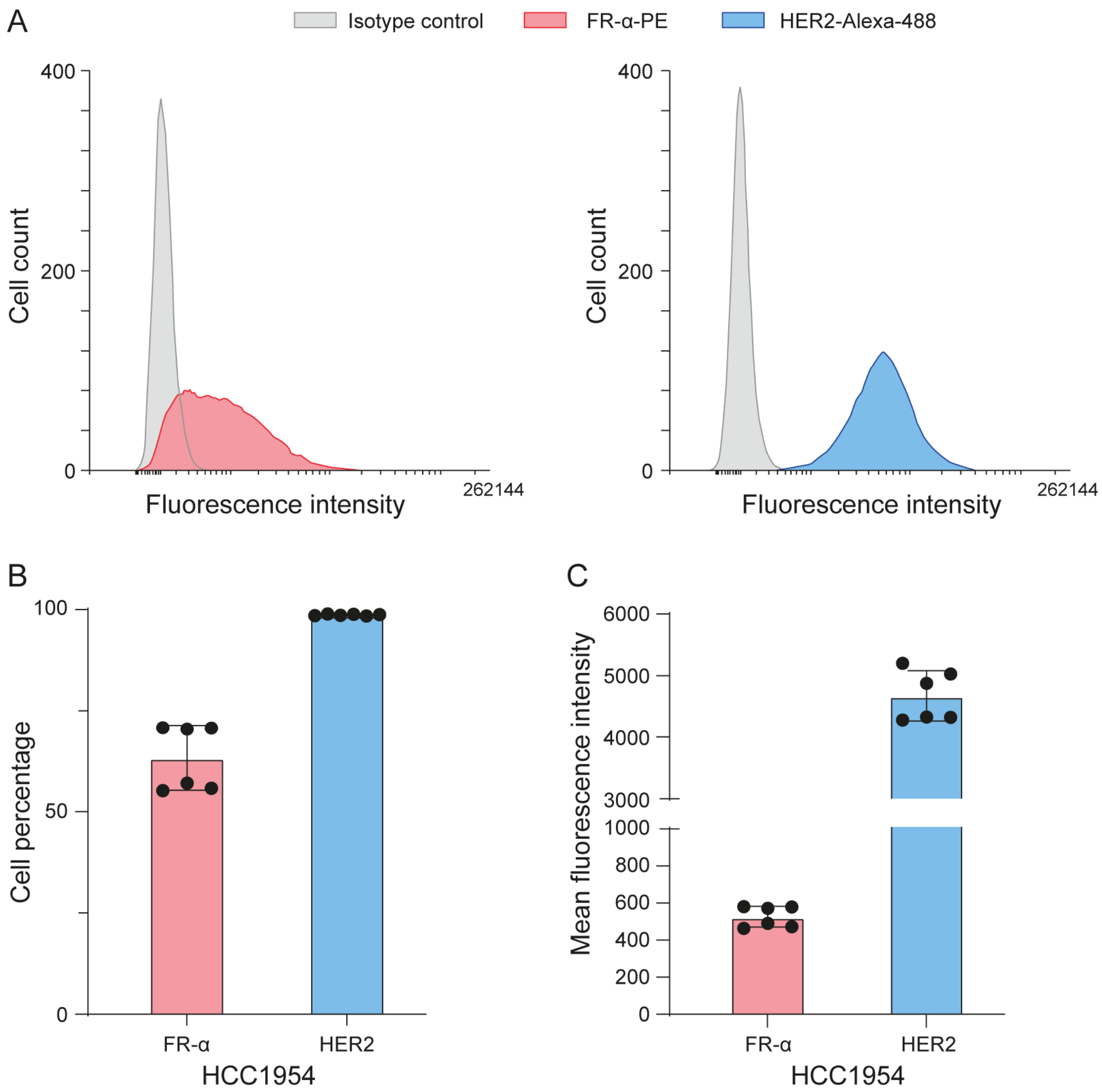

Figure S6. FR-α and HER2 expression on human HCC1954 breast cancer cells. (A) Representative flow cytometry histograms of HCC1954 cells stained with phycoerthrin (PE)-conjugated anti–FR-α (red) and Alexa-488-conjugated anti–HER2 (blue), and corresponding isotype control (grey) antibodies. Quantification of FR-α and HER2 expression presented as (B) percentage and (C) mean fluorescence intensity.

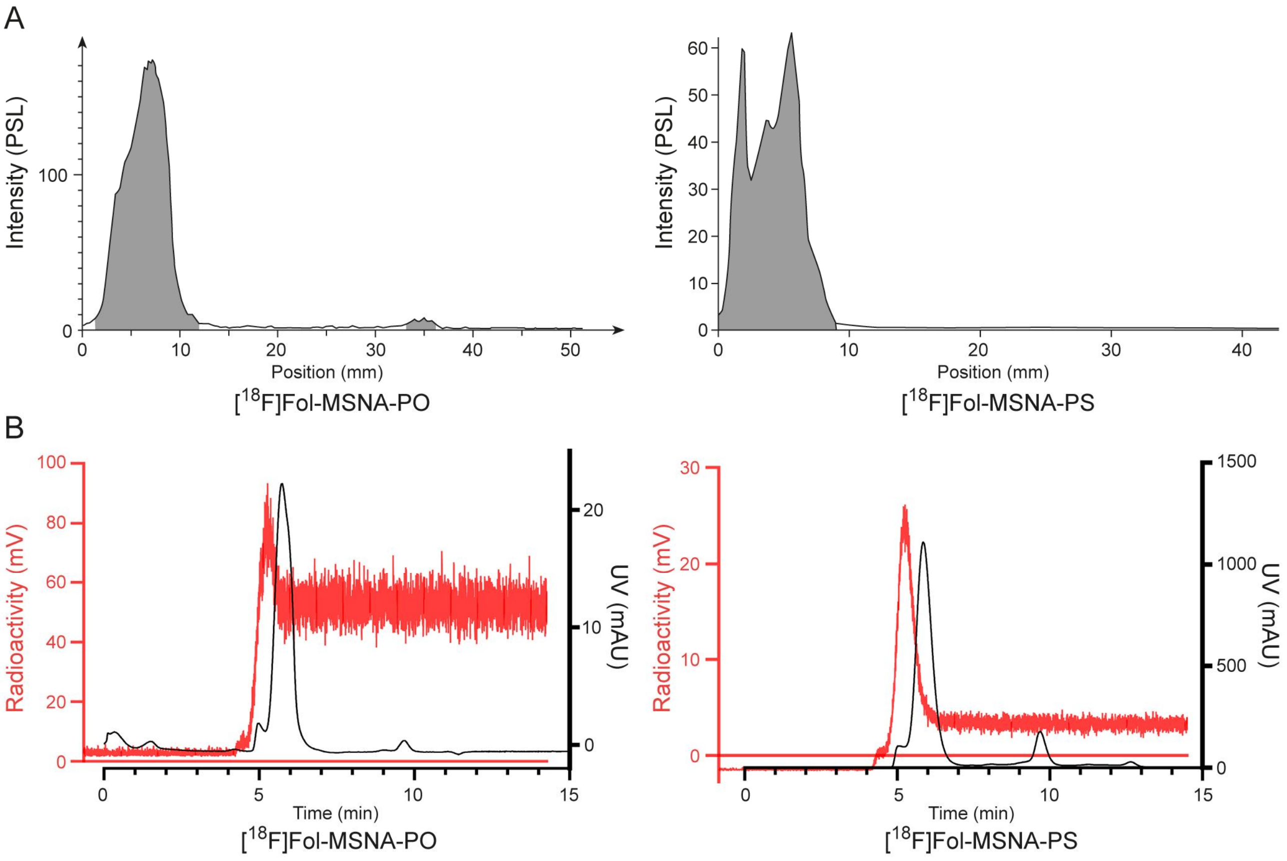

Figure S7. Radio-TLC (A) and radio-SEC (B) of [^{18}F]Fol-MSNA-PO and [^{18}F]Fol-MSNA-PS. The elevated radiodetector signal (red chromatograms on B panels) that remains high after the peak is due to sticking in the tubing. The second UV peak corresponds to single-stranded oligonucleotide caused by partial dissociation of the hybridization complex.

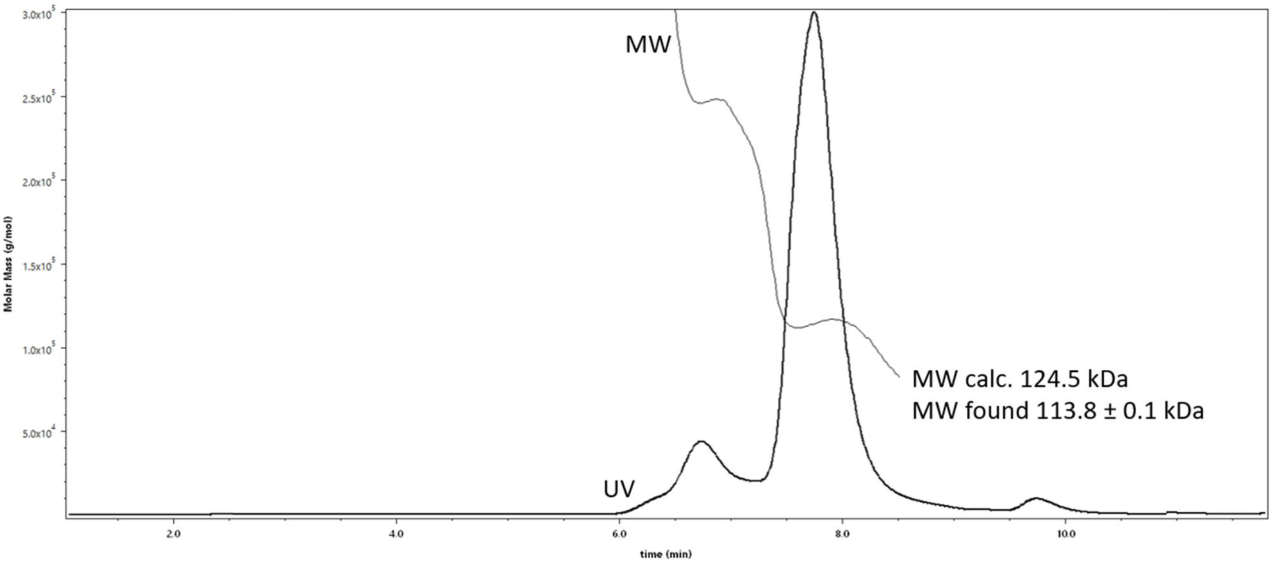

Figure S8. SEC-MALS profile of [^{18}F]Fol-MSNA-PO.

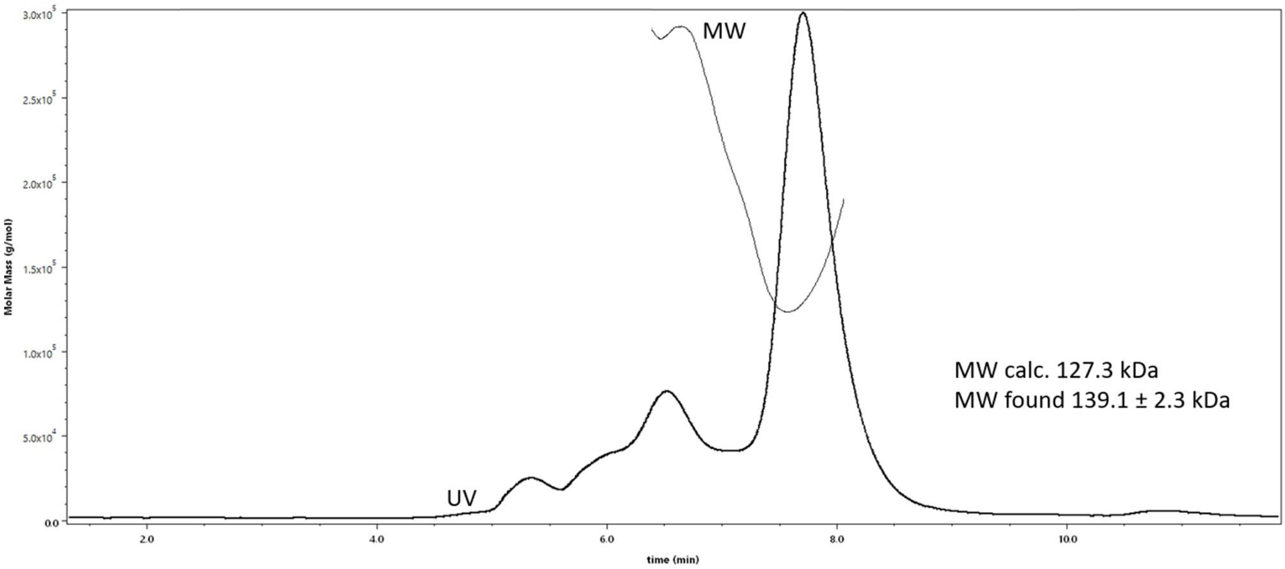

Figure S9. SEC-MALS profile of [18F]Fol-MSNA-PS.

Table S1. Standardized uptake values and statistical analysis of the tumor and tumor-to-muscle ratio at 60 min post-injection of ^{18}F-labeled MSNAs into HCC1954 tumor-bearing female mice.

	[18F]MSNA-PO	[18F]Fol-MSNA-PO	[18F]MSNA-PS	[18F]Fol-MSNA-PS	Statistical significance (*p* value)		
					[18F]MSNA-PO vs. [18F]Fol-MSNA-PO	[18F]MSNA-PS *vs.* [18F]Fol-MSNA-PS	[18F]Fol-MSNA-PO *vs.* [18F]Fol-MSNA-PS
Tumor, SUV	0.16 ± 0.05	0.29 ± 0.03	0.37 ± 0.17	0.17 ± 0.07	** (0.0073)	* (0.0320)	** (0.0095)
Tumor-to-muscle ratio	1.73 ± 0.21	2.17 ± 0.28	2.48 ± 0.78	2.84 ± 0.89	* (0.0181)	ns (0.4965)	ns (0.1691)

[18F]MSNA-PO and [18F]MSNA-PS data are reproduced from a previously published paper.[1] ns = not significant, *$p < 0.05$, **$p < 0.01$.

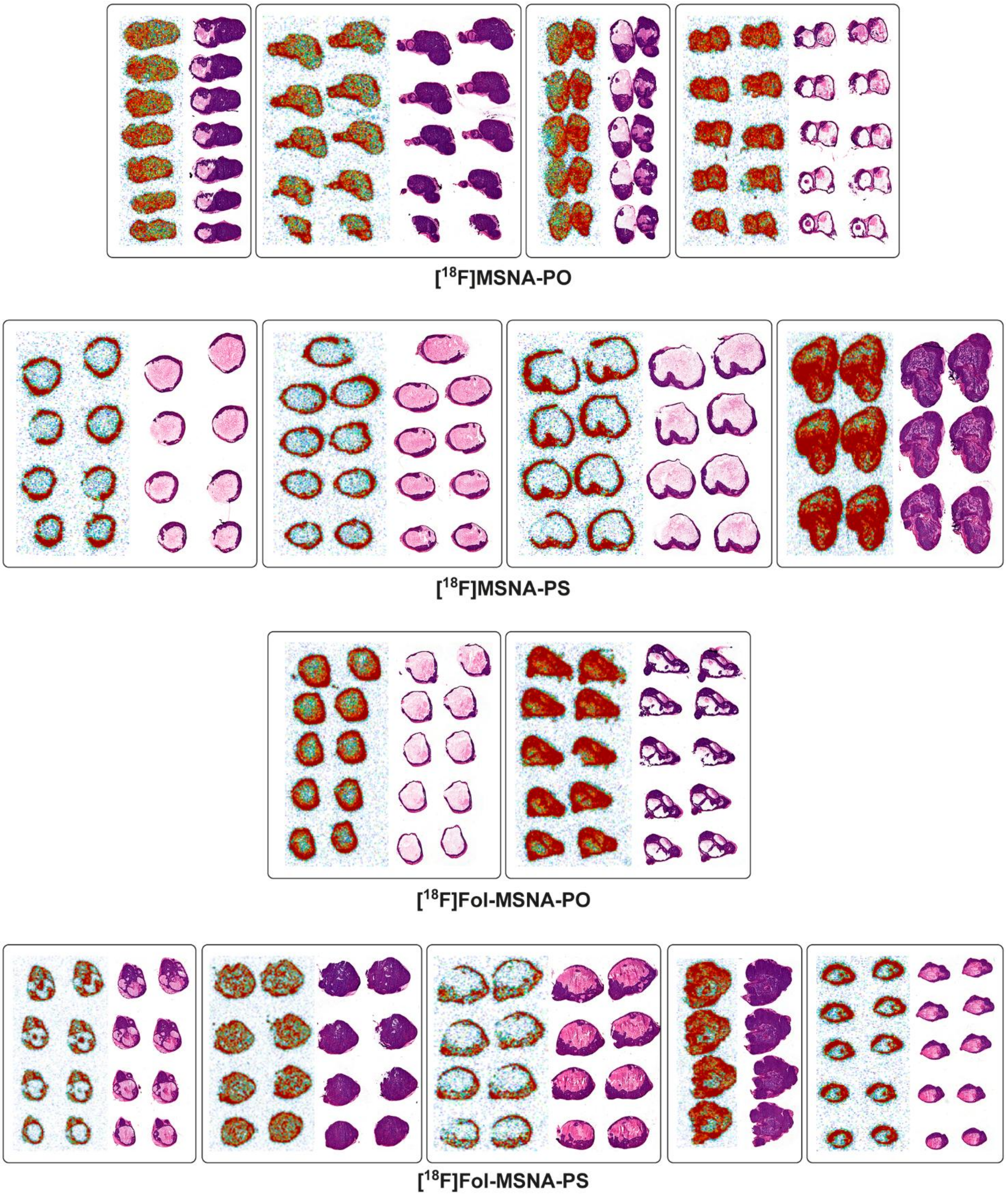

Figure S10. Autoradiography (left panels) of 20 µm tumor cryosections at 60 min post-intravenous administration of ^{18}F-labeled MSNAs into HCC1954 tumor-bearing mice. In hematoxylin-eosin staining (right panels) viable tumor tissue appears blue, while necrotic cells stain pink. [^{18}F]MSNA-PO and [^{18}F]MSNA-PS data are reproduced from a previously published paper.[1]

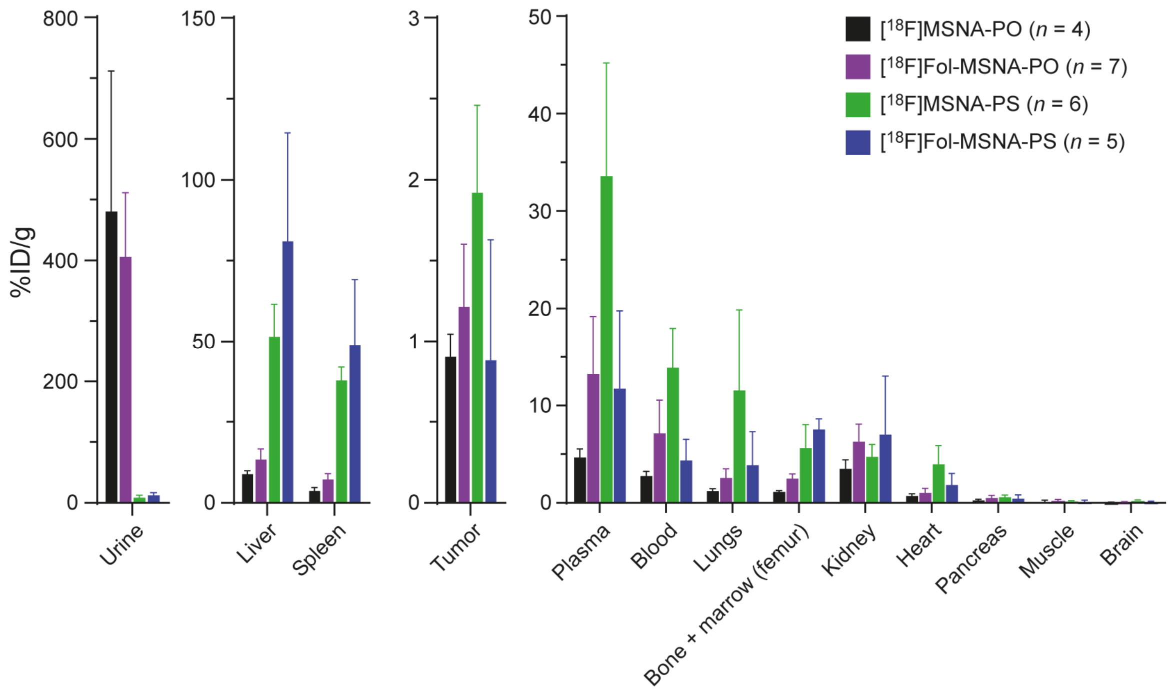

Figure S11. *Ex vivo* biodistribution at 60 min post-intravenous administration ^{18}F-labeled MSNAs into HCC1954 tumor-bearing female mice. [^{18}F]MSNA-PO and [^{18}F]MSNA-PS data are reproduced from a previously published paper.[1]

References

(1) Äärelä, A. A.; Auchynnikava, T.; Moisio, O.; Liljenbäck, H.; Andriana, P.; Iqbal, I.; Lehtimäki, J.; Rajander, J.; Salo, H.; Roivainen, A.; Airaksinen, A. J.; Virta, P. In Vivo Imaging of [60]Fullerene-Based Molecular Spherical Nucleic Acids by Positron Emission Tomography. *Mol. Pharm.* 2023, *20* (10), 5043–5051.
